# Microbial diversity supports nitrification: insights from a full-scale anoxic/oxic wastewater treatment process

**DOI:** 10.1128/aem.01803-25

**Published:** 2025-10-22

**Authors:** Yung-Hsien Shao, Hsiao-Pei Lu, Jer-Horng Wu

**Affiliations:** 1 Department of Environmental Engineering, National Cheng Kung University 34912, Tainan, Taiwan; 2 Department of Biotechnology and Bioindustry Sciences, National Cheng Kung University 34912, Tainan, Taiwan; Georgia Institute of Technology, Atlanta, Georgia, USA

**Keywords:** anoxic/oxic process, microbial diversity, nitrification, microbial association, biodiversity–ecosystem functioning relationship

## Abstract

Nitrification is a crucial process for removing nitrogen in wastewater treatment systems. While previous research has primarily focused on nitrifying microorganisms, the impact of the broader diversity of non-nitrifying microbes on nitrification remains insufficiently explored. This study examined the relationship between microbial diversity and nitrification performance, taking into account the associations with nitrifiers in a full-scale anoxic/oxic activated sludge process. The microbial community was divided into a nitrifier subcommunity, comprising taxa directly responsible for nitrification, and nitrifier-associated subcommunities, comprising taxa that potentially interact with nitrifiers. Notably, the nitrifier-associated subcommunity consistently exhibited greater explanatory power for variations in nitrification rates than both the total microbial community and the nitrifier subcommunity, regardless of environmental and feeding conditions. Furthermore, the explanatory power of the nitrifier-associated subcommunity increased with stricter association cutoffs, with a core set of 13 taxa identified at an association cutoff of 0.6, accounting for up to 33% of the observed variations in the nitrification rate. Random forest ranked the dynamics of the core nitrifier-associated subcommunity among the top 3 most important variables out of 30 environmental and microbial factors, collectively explaining 82% of the variation in the nitrification rate. These findings underscore the importance of broader microbial diversity—beyond the diversity of nitrifiers alone—in supporting nitrification performance, highlighting the contribution of non-nitrifying microbes through their associations with nitrifiers. This study offers novel insights into the relationship between biodiversity and nitrification, providing valuable implications for managing microbial communities to optimize nitrogen removal in wastewater treatment ecosystems.

**IMPORTANCE:**

Nitrification is essential for removing nitrogen from wastewater. While most research has concentrated on the nitrifiers directly responsible for this process, the role of the broader microbial community in influencing nitrification has been less explored. This study reveals a previously unrecognized relationship: the diversity of microbial taxa associated with nitrifiers explains more variation in nitrification rates than the diversity of either the total microbial community or the nitrifiers alone in a real-world wastewater treatment plant. By demonstrating the influential role of the diversity of the broader microbial community in shaping nitrification performance, this work introduces a new perspective on the ecological drivers of nitrification dynamics beyond nitrifier functional diversity. This insight provides a foundation for better microbial community management to achieve stable and efficient nitrogen removal. Our results deepen current understanding of the ecological complexity underpinning wastewater treatment processes, stressing the importance of microbial diversity in wastewater treatment system functionality.

## INTRODUCTION

Activated sludge processes are widely employed in wastewater treatment plants (WWTPs). The processes rely on collective metabolic activities of complex microbial communities to remove dissolved organic matter and nutrients from wastewater. Recent technological advancements, including amplicon sequencing and meta-omics, have considerably enhanced the understanding of microbiomes in WWTPs (1). A global survey estimated that bacterial richness in activated sludge worldwide reaches approximately 10⁹ species (2). Despite this vast diversity, the impact of microbial diversity on the functional performance of activated sludge ecosystems remains poorly understood.

The relationship between microbial diversity and ecosystem function has been the central focus of research across various ecosystems, including soil (3, 4), marine (5), and forest ecosystems (6). Microbial diversity has been shown to affect natural ecosystem functioning positively. Most studies on the topic of microbial diversity have typically emphasized “broad” processes involving diverse microbial taxa (7), such as respiration and biomass production (4, 8, 9). By contrast, WWTP operators employ process controls, including sludge retention time, food-to-microorganism ratio, and aeration, to regulate complex microbial communities and ensure that specific functional groups effectively perform specialized biochemical processes, such as nitrification, denitrification, anammox, and methanogenesis. These processes, performed exclusively by specific microbial groups, are categorized as “narrow” processes (7). Studies of WWTP ecosystems have predominantly explored the relationships between specific functions and the diversity of microbial populations responsible for these functions—referred to as functional diversity (10, 11). In contrast, the influence of emergent properties of the broader microbial communities, such as biodiversity and community dynamics, has been largely overlooked (12).

The anoxic/oxic (AO) activated sludge process is widely used for treating organic matter and inorganic nitrogen in wastewater. This process removes nitrogenous pollutants in two stages. Ammonia is first oxidized to nitrate through nitrification in oxic tanks. A portion of the oxic tank effluent is then recirculated to the anoxic tanks, where nitrate is reduced to nitrogen gas through denitrification. While nitrification is a critical step in the AO process, maintaining nitrification stability in WWTPs is challenging (13). Nitrification is mediated by restricted groups of autotrophic microorganisms, including ammonia-oxidizing bacteria (AOB), ammonia-oxidizing archaea (AOA), complete ammonia oxidation (comammox) bacteria*,* and nitrite-oxidizing bacteria. As a “narrow” process, nitrification performance is typically assumed to be influenced by these specific functional groups—collectively referred to as nitrifiers—rather than by the overall microbial community, which is predominantly composed of heterotrophs. Studies have reported that nitrification performance is more strongly correlated with the diversity of nitrifiers than with the diversity of overall microbial communities (14, 15). However, focusing solely on the functional diversity of nitrifiers may provide an incomplete understanding of the relationship between the microbial community and nitrification, as the broader microbial community may indirectly influence the stability and efficiency of nitrification.

Nitrifiers typically constitute only a small fraction of the total microbial community in real-world WWTPs and coexist with a wide variety of other microorganisms (16–18). The often-reported weak correlation between overall microbial diversity and nitrification performance may, to some extent, be attributed to the presence of numerous “noise” microbes—taxa that neither directly participate in nor indirectly influence nitrification processes (3). However, network-based analyses of microbial co-occurrence patterns have revealed significant associations between nitrifiers and coexisting microbial taxa in WWTPs (18, 19), suggesting potential ecological interactions. Based on these ecological roles, the microbial community can thus be categorized into three groups: (i) nitrifiers that directly mediate nitrification, (ii) non-nitrifying microbes that interact with nitrifiers, and (iii) microbes that neither participate in nitrification nor interact with nitrifiers. This third group may represent the “noise” that obscures clear relationships between community diversity and nitrification performance. Therefore, taking into account the interactions between nitrifiers and their coexisting taxa may help gain a clearer understanding of how microbial diversity affects nitrification performance.

**Fig 1:**
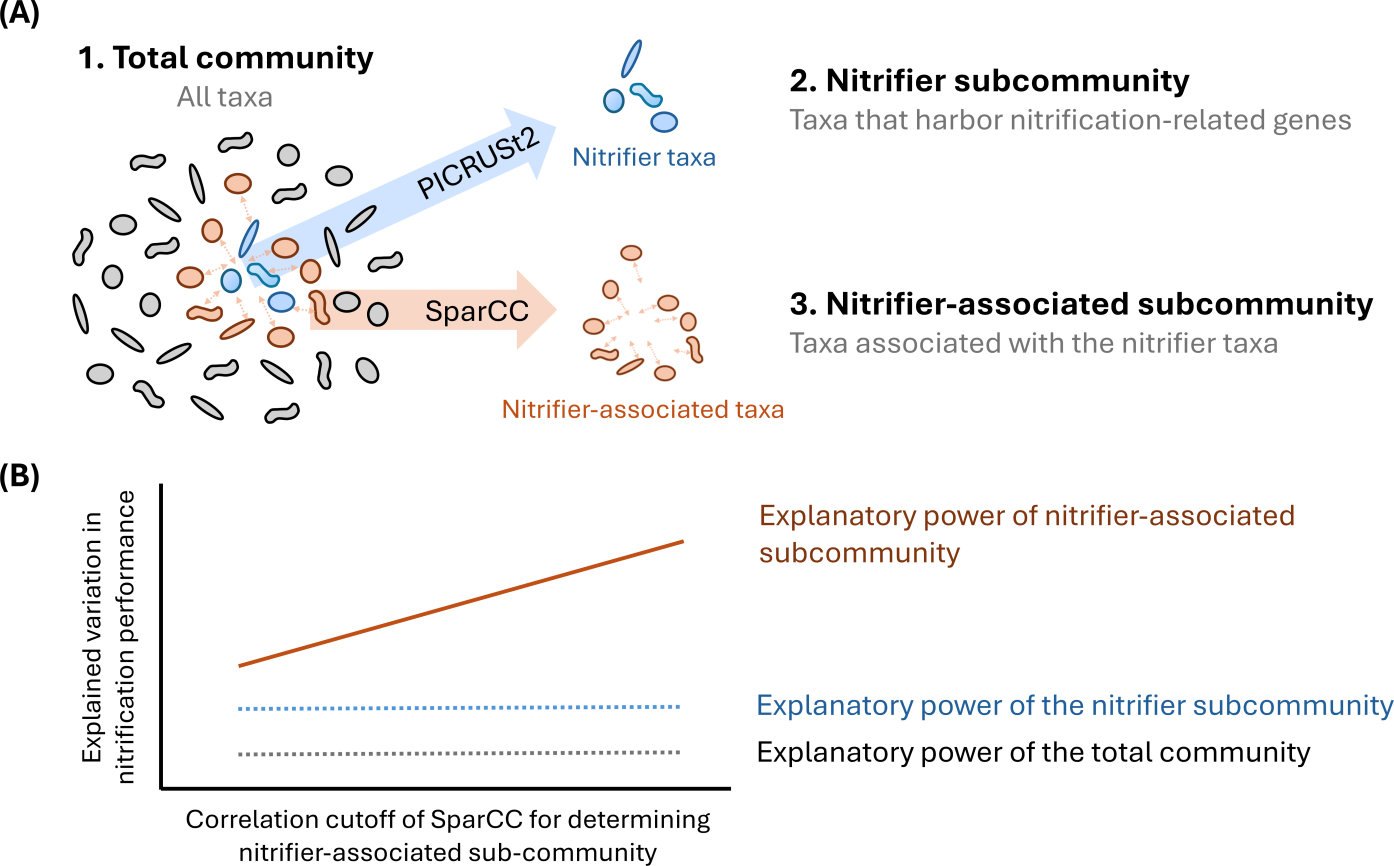
Definition of total microbial community and subcommunities, and study hypothesis. (**A**) The total community comprises all detected taxa. Taxa predicted to harbor nitrification-related genes, as identified using PICRUSt2, were selected to form the nitrifier subcommunity. Subsequently, taxa associated with nitrifiers were identified using the SparCC method to form the nitrifier-associated subcommunities. (**B**) The study hypothesized that filtering out noise taxa on the basis of their associations with nitrifier K-mer taxonomic units would enhance the ability of community diversity to explain variations in nitrification performance. To test this hypothesis, nitrifier-associated subcommunities were constructed using a range of correlation cutoffs from 0.1 to 0.9 in the SparCC analysis. The alpha (species richness and abundance) and beta diversity (compositional dissimilarities between communities) of these communities were then calculated, and their relationships with nitrification performance were examined using multiple linear regression.

In this study, we conducted daily sampling at a full-scale industrial WWTP and divided the total community, which includes all identified taxa, into two types of subcommunities (Fig. 1A). First, we used PICRUSt2 (20) to identify taxa harboring nitrification-related genes, thereby defining a nitrifier subcommunity composed of taxa directly involved in nitrification. The diversity of this functional group is expected to influence nitrification performance significantly (14). Next, we constructed microbial co-occurrence networks using the SparCC method (21) to identify taxa that associate with nitrifying bacteria, forming the nitrifier-associated subcommunity. We hypothesized the following: (i) the diversity of the nitrifier-associated subcommunity would explain a greater proportion of the variation in nitrification performance compared with the diversity of the total community, and (ii) stronger associations would enhance the explanatory power of the diversity of the nitrifier-associated subcommunity in predicting nitrification performance (Fig. 1B). To test these hypotheses, we analyzed alpha diversity (species richness and abundance, hereafter referred to as community diversity) and beta diversity (compositional dissimilarities between communities) for the total community and subcommunities. Multiple linear regression was then applied to quantify the relationships between these factors and nitrification performance. The findings revealed that compositional changes of the nitrifier-associated subcommunity exhibited a stronger correlation with nitrification performance than did either the total community or the nitrifier subcommunity, supporting our hypothesis that the diversity of the broader microbial community—beyond just the functional diversity of nitrifiers—plays a significant role in influencing nitrification performance. These findings offer novel insights into the relationship between biodiversity and nitrification, thereby contributing to the predictive management of nitrification in WWTP ecosystems.

## RESULTS

### Nitrification performance of the AO process

The full-scale petrochemical WWTP in the study employed the AO process to remove organic carbon and nitrogen from acrylonitrile-butadiene-styrene (ABS) resin manufacturing wastewater (Fig. S1). The influent wastewater had a chemical oxygen demand (COD) of approximately 2,500 mg/L and a total nitrogen (TN) concentration exceeding 100 mg-N/L. Nitrogen removal was achieved through denitrification in the anoxic tank, which received raw wastewater and nitrate-rich supernatant recycled from the oxic tanks, where ammonia was oxidized to nitrate. Organic carbon in the raw wastewater was metabolized either through aerobic oxidation in the oxic tanks or as an electron donor for anaerobic respiration in the anoxic tanks.

Two separate data sets were collected, and substantial variations in wastewater characteristics were observed between Periods 1 and 2. More than half of the measured environmental parameters significantly differed between the two periods, including concentrations of inorganic carbon (IC), ammonium, nitrite, TN, and mixed liquor suspended solids, as well as pH, temperature, and the flow rate of settled sludge returned to the A1 tank (Table 1). The temporal dynamics of these parameters are shown in Fig. S2. The AO process received influent wastewater containing TN concentrations fluctuating between 50 and 200 mg-N/L, with ammonium and organic nitrogen as the primary nitrogen pollutants. Notably, TN concentrations in the influent significantly increased from 110.5 ± 26.1 mg-N/L in Period 1 to 134.4 ± 28.3 mg-N/L in Period 2. This increase was primarily attributable to a higher ammonium concentration in Period 2 (70.7 ± 29.6 mg-N/L) than in Period 1 (44.4 ± 12.9 mg-N/L). In particular, the influent ammonium concentration increased to 87.6  ±  24.0  mg-N/L during early Period 2 (days 78–106), which was approximately twofold higher than that in late Period 2 (44.9  ±  15.3  mg-N/L, days 107–126) and in Period 1. Organic nitrogen concentrations remained relatively stable between the two periods. The COD to TN ratio was higher in Period 1 (23.4 ± 6.1) than in Period 2 (19.7 ± 4.3). Moreover, the concentration of IC, the carbon source for nitrifying bacteria, decreased from 92.5 ± 12.5 mg/L in Period 1 to 62.7 ± 7.0 mg/L in Period 2. Throughout both periods, the dissolved oxygen concentration and pH in oxic tanks were maintained at approximately 5 mg/L and 7, respectively, ensuring sufficient electron acceptors and neutral conditions for nitrification.

**TABLE 1:** Summary statistics of environmental variables, including influent characteristics and reactor operational parameters^*a*^

Variable^*b*^	Value in Period 1 (*n* = 44) (mean ± SD)	Value in Period 2 (*n* = 48) (mean ± SD)	Test	*P*-value
COD (mg/L)	2,462.7 ± 332.8	2,561.5 ± 386.7	*t*-test	0.189
TOC (mg/L)	629.4 ± 73.4	664.2 ± 116.3	*t*-test	0.087
IC (mg/L)	**92.5 ± 12.5**	**62.7 ± 7.0**	*t*-test	**0.000**
Ammonium (mg-N/L)	**44.4 ± 12.9**	**70.7 ± 29.6**	*t*-test	**0.000**
Nitrite (mg-N/L)	**0.3 ± 0.4**	**0.7 ± 0.5**	**Wilcoxon**	**0.000**
Nitrate (mg-N/L)	0.7 ± 0.3	0.6 ± 0.3	Wilcoxon	0.191
Organic-N (mg-N/L)	65.2 ± 25.3	66.6 ± 25.8	*t*-test	0.876
TN (mg-N/L)	**110.5 ± 26.1**	**134.4 ± 28.3**	*t*-test	**0.000**
Conductivity (μS/cm)	3,239.0 ± 357.3	3,252.3 ± 594.5	*t*-test	0.981
Salinity (^0^/_00_)	1.7 ± 0.2	1.7 ± 0.3	Wilcoxon	0.753
TDS (mg/L)	2,106.8 ± 230.8	2,114.7 ± 386.6	*t*-test	0.989
Flowrate (m^3^/day)	3,141.8 ± 236.2	2,925.0 ± 491.7	Wilcoxon	0.125
MLSS (mg/L)^*c*^	**4,228.8 ± 329.1**	**3,907.1 ± 769.7**	**Wilcoxon**	**0.000**
pH^*c*^	**7.14 ± 0.08**	**7.11 ± 0.09**	**Wilcoxon**	**0.045**
DO (mg/L)^*c*^	5.6 ± 0.1	5.2 ± 0.6	Wilcoxon	0.592
Temperature (°C)^*c*^	**34.4 ± 1.5**	**31.8 ± 1.1**	**Wilcoxon**	**0.000**
Return sludge MLSS (mg/L)	7,550.5 ± 762.0	7,162.8 ± 2,307.6	Wilcoxon	0.094
Return flow rate (m^3^/day)^*d*^	**4,675.6 ± 505.9**	**3,341.0 ± 1,170.5**	**Wilcoxon**	**0.000**

^*a*^ Normality of each variable within each period was evaluated using the Shapiro-Wilk test. Depending on the results, between-period differences were tested with either an independent *t*-test (when both periods were normally distributed) or a Wilcoxon rank-sum test (when normality was not satisfied). Variables with significant differences between Period 1 and Period 2 (*P* < 0.05) are highlighted in bold.

^*b*^ TOC, total organic carbon; organic-N (organic nitrogen); TDS, total dissolved solids; MLSS, mixed liquor suspended solids; DO, dissolved oxygen.

^*c*^ MLSS, pH, DO concentration, and temperature were measured in the fourth aerobic tank.

^*d*^ Flow rate of settled sludge returned to the A1 tank.

**Fig 2:**
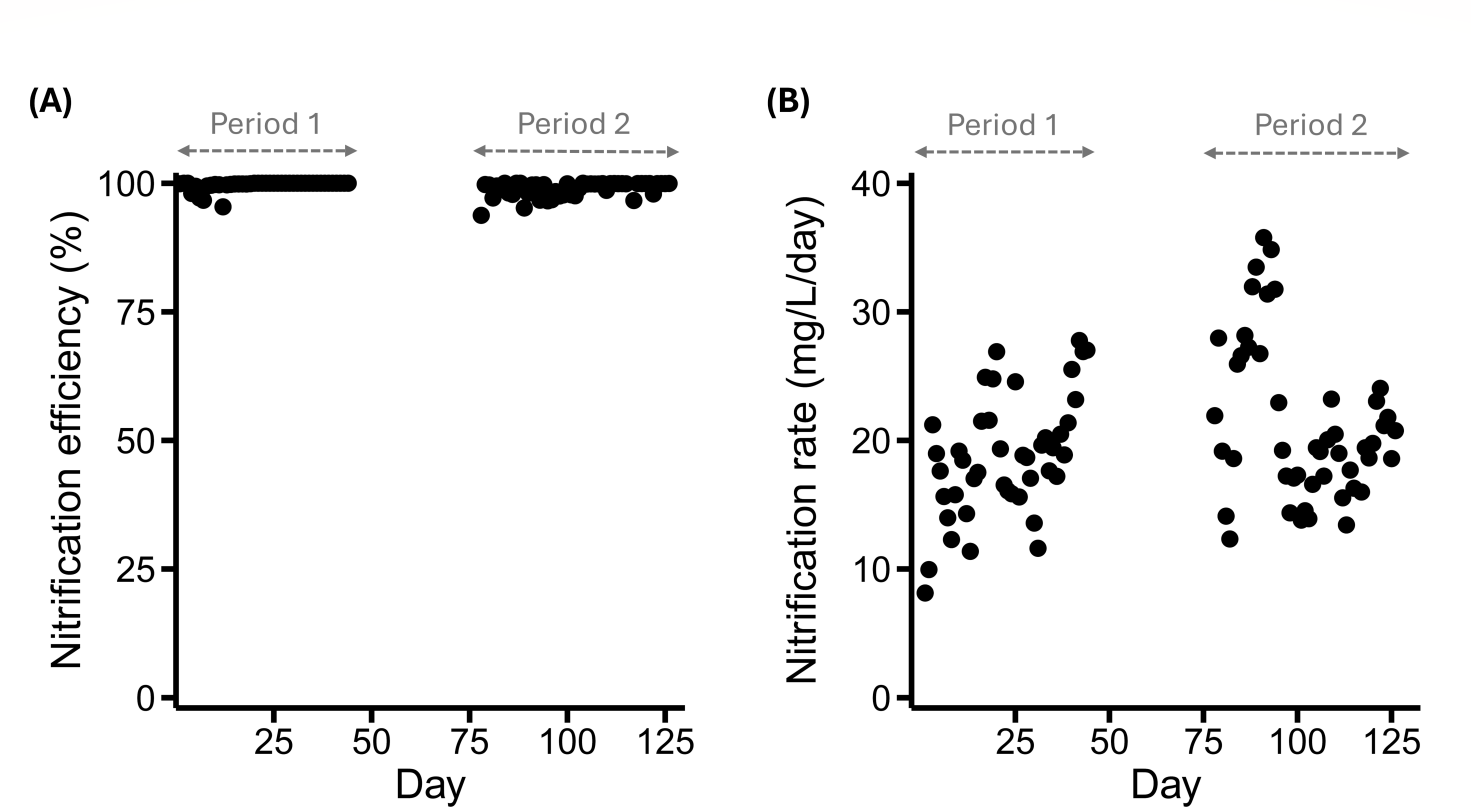
Nitrification performance of WWTP. (**A**) Nitrification efficiency. (**B**) Nitrification rate. Nitrate production was first calculated as the difference between the sum of organic nitrogen, NH₄^+^-N, and NO₂⁻-N in the influent and effluent. Nitrification efficiency was then determined by dividing nitrate production by the sum of influent organic nitrogen, NH₄^+^-N, and NO₂⁻-N. Nitrification rate was calculated by dividing nitrate production by the hydraulic retention time. Nitrification rate was then chosen as an indicator of the community’s nitrification capability, as it reflects the amount of ammonium converted to nitrate per day. This study did not conduct sampling for days 45–77.

Despite the distinct wastewater characteristics observed in the two periods, the AO process exhibited robust performance, achieving COD removal efficiency exceeding 95% and nitrogen removal of approximately 90%. Moreover, the microbial community maintained consistently high nitrification efficiency, exceeding 95% in both periods (Fig. 2A), with nitrification rates varying between 8 and 36 mg-N/L/day (Fig. 2B). These results highlight not only the high treatment efficiency and operational stability of the AO process but, more importantly, the remarkable resilience and adaptability of the microbial community in sustaining effective nitrification despite fluctuations in nitrogen loads and compositions. Therefore, we further explored how community diversity supports the robustness of nitrification in the AO process.

### Relationship between diversity and nitrification

**Fig 3:**
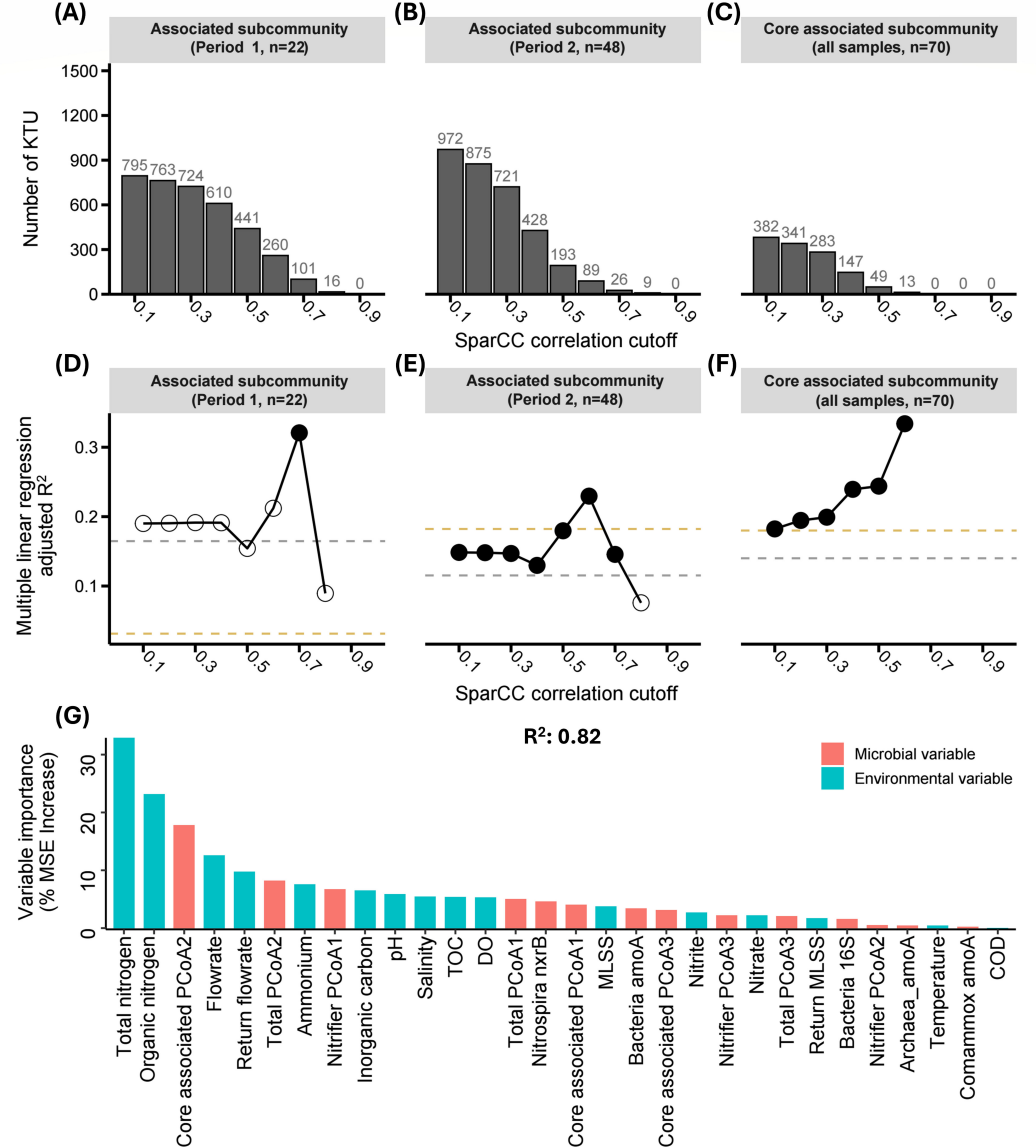
Nitrifier-associated KTUs and relationships between dynamics of nitrifier-associated subcommunities and nitrification rate. Nitrifier-associated subcommunities for each period were separately constructed using data from Periods 1 and 2. KTUs consistently associated with nitrifiers across both periods were defined as core nitrifier-associated KTUs. Bar plots display the number of KTUs across different correlation coefficient cutoffs ranging from 0.1 to 0.9 in the (**A**) nitrifier-associated subcommunity in Period 1, (**B**) nitrifier-associated subcommunity in Period 2, and (**C**) core nitrifier-associated subcommunity. The proportion of variation in nitrification rate explained by the dynamics of the (**D**) nitrifier-associated subcommunity in Period 1, (**E**) nitrifier-associated subcommunity in Period 2, and (**F**) core nitrifier-associated subcommunity across all samples, using the first three axes of principal coordinate analysis derived from Bray-Curtis dissimilarity as predictors in multiple linear regression models. Hollow circles indicate regression models with *P-*values of ≥0.05, and solid circles indicate *P*-values of <0.05. The gray dashed line represents the variation explained by the total microbial community, and the yellow dashed line represents the variation explained by the nitrifier subcommunity. (**G**) The relative importance of environmental and microbial variables in explaining the nitrification rate, as determined by a random forest regression model with an *R*^2^ of 0.82.

To investigate the relationship between microbial diversity and nitrification, we defined three types of microbial communities in the AO process (Fig. 1A). A total of 22,712 amplicon sequence variants (ASVs) were detected across all samples (*n* = 70). These ASVs were then re-clustered into 1,886 KTUs (K-mer taxonomic units), representing the total community. On average, 12.0 ± 10.4 ASVs were re-clustered into a KTU, with a sequence similarity of 99.6 ± 1.0%. By reducing redundancy and incorporating taxonomic relatedness among closely related ASVs, KTUs offer a biologically meaningful alternative to ASVs, enhancing both statistical robustness and ecological interpretability in relation to biotic and abiotic factors (22). The nitrifier subcommunity was constructed by selecting KTUs predicted to harbor *amo* or *nxr* genes, followed by phylogenetic verification to ensure alignment with known nitrifiers. Microbial association networks were separately constructed for Periods 1 and 2, and KTUs associated with nitrifiers were selected to form the nitrifier-associated subcommunities. Additionally, nitrifier-associated KTUs across both periods were defined as core nitrifier-associated KTUs. Details of the KTU re-clustering results for nitrifier and core nitrifier-associated KTUs are presented in Table S1. Next, we adjusted the correlation coefficient cutoffs in the SparCC analysis to generate multiple nitrifier-associated subcommunities with association thresholds ranging from 0.1 to 0.9 at a significance level <0.01. Only KTUs exhibiting correlations with nitrifiers exceeding the established cutoff were retained in each subcommunity. As the correlation cutoff increased from 0.1 to 0.8, the number of nitrifier-associated KTUs decreased from 795 to 16 in Period 1 (Fig. 3A) and from 972 to 9 in Period 2 (Fig. 3B). By contrast, no KTU was correlated with nitrifiers with a cutoff of ≥0.9 in either period. The number of core nitrifier-associated KTUs also decreased from 382 at a cutoff of 0.1 to 13 at a cutoff of 0.6, with no KTU maintaining an association strength greater than 0.7 with nitrifiers across both periods (Fig. 3C).

The relationship between microbial community and nitrification performance was evaluated using multiple linear regression. For community compositional changes, the nitrifier-associated subcommunity exhibited the highest explanatory power for variations in the nitrification rate at a correlation cutoff of 0.7 in Period 1 (adjusted *R*^2^ = 0.32, *P* < 0.05) and 0.6 in Period 2 (adjusted *R*^2^ = 0.23, *P* < 0.05; Fig. 3D and E). These values surpassed the explanatory power of compositional changes for both the total community (adjusted *R*^2^ = 0.16, *P* > 0.05 in Period 1; adjusted *R*^2^ = 0.12, *P* < 0.05 in Period 2) and the nitrifier subcommunity (adjusted *R*^2^ = 0.03, *P* > 0.05 in Period 1; adjusted *R*^2^ = 0.18, *P* < 0.05 in Period 2). Additionally, the explanatory power of compositional changes for the nitrifier-associated subcommunities declined when correlation cutoffs exceeded the optimal thresholds, which may be attributed to a reduction in the number of retained nitrifier-associated KTUs (Fig. 3A and B). Similarly, models based on the core nitrifier-associated subcommunity demonstrated superior predictive capacity compared with those based on the total and nitrifier subcommunities, with adjusted *R*^2^ values of 0.14 and 0.18 (both *P* < 0.05), respectively (Fig. 3F). The explanatory power of compositional changes for the core nitrifier-associated subcommunity increased with higher correlation cutoffs, peaking at a cutoff of 0.6, where it accounted for up to 33.3% of the variation in nitrification rate across all samples.

Because changes in microbial community composition may take time to respond to variations in environmental conditions, we further examined the effect of time-lag treatment on the relationship between the compositional changes of the core nitrifier-associated subcommunity and the nitrification rate. Time lags of 2, 4, 6, and 8 days were applied to the compositional changes of the core nitrifier-associated subcommunity, corresponding to the hydraulic retention time of the WWTP (approximately 5–8 days). As shown in Fig. S3A, when a 2 day time lag was applied to the compositional changes of the core nitrifier-associated subcommunity, the relationship with the nitrification rate remained similar to that without a time lag (Fig. 3F). However, as the time lag interval increased, the explanatory power of the core nitrifier-associated subcommunity composition declined. With an 8-day time lag, the compositional changes in all communities showed no significant correlation with the nitrification rate (Fig. S3D). This finding is likely due in part to the extensive internal recirculation from the fourth oxic tank to the first anoxic tank at a flow rate seven times that of the influent, which can rapidly distribute environmental changes throughout the system.

Next, we investigated the relationship between community diversity and nitrification rate. Diversity in the nitrifier subcommunity failed to explain variations in nitrification rate, with adjusted *R*^2^ values <0.1 across all models (Table 2). The explanatory power of the total community varied, with adjusted *R*^2^ values of −0.03 in Period 1, 0.22 in Period 2, and 0.11 across all samples. By contrast, diversity in the nitrifier-associated subcommunities, determined using their respective optimized correlation cutoffs, consistently explained 20%–21% of the variation in nitrification rate, demonstrating a stable predictive capacity for nitrification performance. However, the explanatory power of diversity in the nitrifier-associated subcommunities was consistently lower than that of community composition, when assessed using their respective optimized cutoffs, across Periods 1 and 2, and all samples (Fig. 3; Table 2). This suggests that variations in community diversity in nitrifier-associated subcommunities have a relatively smaller effect on the nitrification rate than does community composition. Additionally, random forest regression identified the dynamics of the core nitrifier-associated subcommunity as among the top 3 important variables out of 30 environmental and microbial factors, collectively explaining 82% of the variation in the nitrification rate (Fig. 3G). These findings underscore the critical role of microbial population dynamics in specific subsets of the microbial community, particularly the KTUs that are consistently associated with nitrifiers, in influencing nitrification rates in the full-scale AO process.

**TABLE 2:** Alpha diversity of the total community and subcommunities explained the variation in nitrification rate through multiple linear regression analysis^*b*^

Community	Predictor	Nitrification rate (Period 1, *n* = 22)		Nitrification rate (Period 2, *n* = 48)		Nitrification rate (all samples, *n* = 70)	
		Coefficient	Adj*R*^2^	Coefficient	Adj*R*^2^	Coefficient	Adj*R*^2^
Total community	Richness	0.22	−0.03	**1.92**	**0.22**	**0.81**	**0.11**
	Shannon	0.23		**−3.05**		**−1.43**	
	Simpson	−0.88		**1.03**		**0.49**	
Nitrifier subcommunity	Richness	0.23	0.003	−0.10	0.05	−0.17	0.02
	Shannon	−0.78		−1.20		0.19	
	Simpson	0.35		1.09		−0.31	
Nitrifier-associated subcommunity^*a*^	Richness	0.30	0.21	**1.49**	**0.20**	**0.55**	**0.21**
	Shannon	1.00		**−2.33**		**−1.75**	
	Simpson	−1.67		**0.89**		**1.14**	

^*a*^ For nitrifier-associated subcommunities, models were constructed across various SparCC correlation coefficient cutoffs, and those with the highest adjusted *R*^2^ values are presented. Specifically, the optimized correlation cutoff for the nitrifier-associated subcommunity in Period 1 was 0.4, for the nitrifier-associated subcommunity in Period 2 was 0.1, and for the core nitrifier-associated subcommunity was 0.4.

^*b*^ Values with significant *P*-values (<0.05) are highlighted in bold.

We also examined the relationship between community compositional change and COD removal rate. The results revealed that the dynamics of all studied communities were marginally associated with COD removal rates, as indicated by adjusted *R*^2^ values of <0.06 across all models (Table S2). This finding suggests that the changes in diversity and composition of nitrifier-associated subcommunities did not significantly influence the COD removal performance in the AO process.

### Microbial community composition

**Fig 4:**
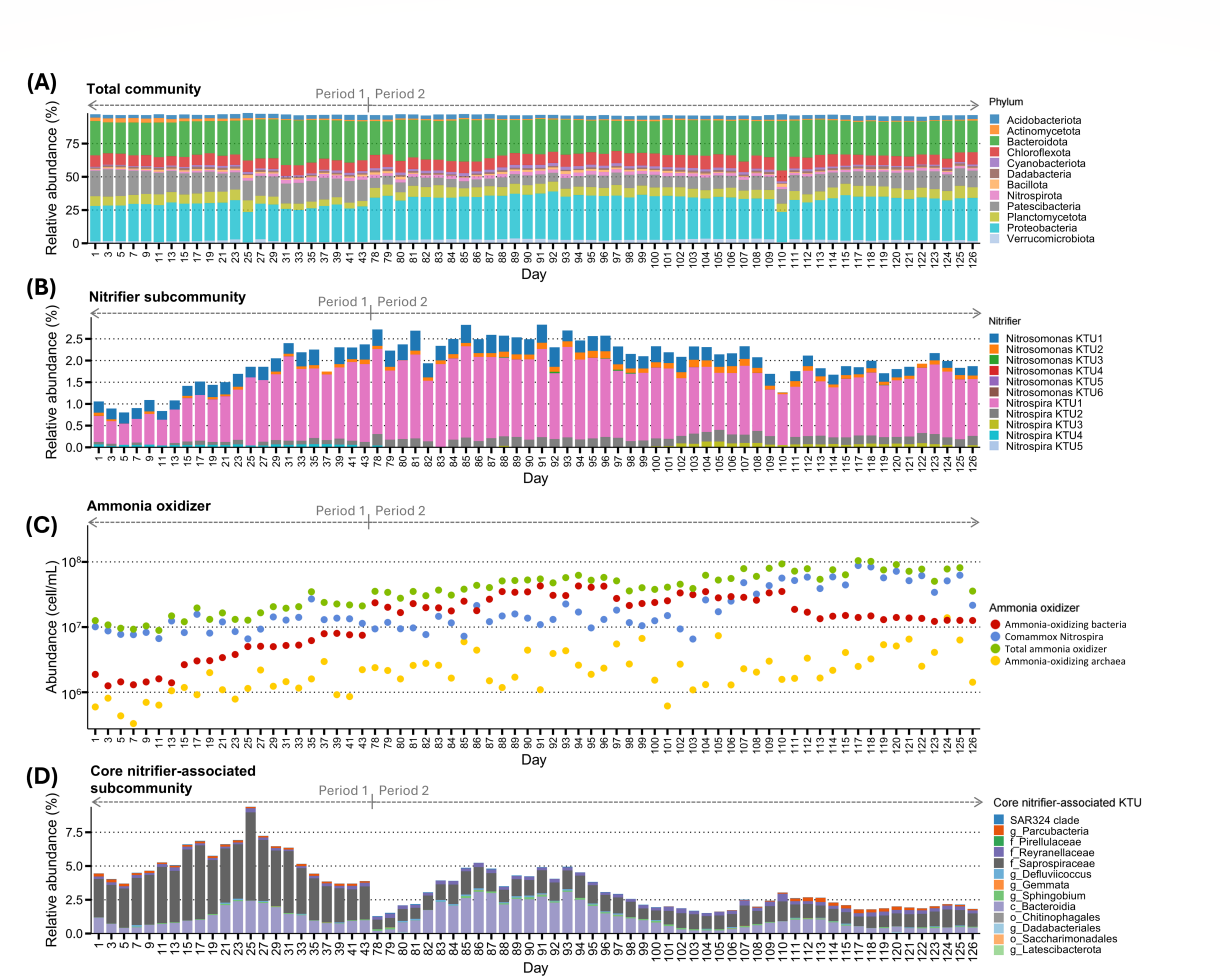
Community composition of the total community and its subcommunities, as well as the abundance of ammonia oxidizers. (**A**) Composition of the total community at the phylum level, displaying only phyla with an average abundance >1%. (**B**) Composition of the nitrifier subcommunity at the KTU level. (**C**) Abundance of ammonia oxidizers. (**D**) The composition of the core nitrifier-associated subcommunity at a correlation cutoff of 0.6, shown at the KTU level. The phylogenetic classification of each KTU in the core nitrifier-associated subcommunity at the highest taxonomic resolution is provided in the legend.

Figure 4A presents the temporal variation in the composition of the total community, displaying only phyla with relative abundances exceeding 1%. The system was consistently dominated by the phyla *Proteobacteria* and *Bacteroidota*, which accounted for more than 50% of the total community across Periods 1 and 2.

Eleven nitrifier KTUs were detected for the nitrifier subcommunity, with six belonging to the genus *Nitrosomonas* and five to the genus *Nitrospira*. Both genera are commonly found in WWTPs (23). These nitrifier KTUs accounted for a small proportion (<3%) of the total community (Fig. 4B). Among them, *Nitrospira* KTU1 was the most abundant throughout the sampling periods. To further characterize the abundance of nitrifiers, a quantitative polymerase chain reaction was performed to quantify the absolute abundance of nitrification-related genes (Fig. S4). To estimate the total abundance of ammonia oxidizers in the WWTP, we calculated the cell abundances of AOA, AOB, and comammox *Nitrospira* by normalizing their respective *amoA* gene abundances based on average gene copy numbers per genome: one copy for AOA *amoA* (24), 2.5 copies for *Nitrosomonas amoA* (25), and one copy for comammox *amoA* (26) (Fig. 4C). The total ammonia oxidizer abundance was then obtained by summing the abundances of AOA, AOB, and comammox *Nitrospira*. In Period 2, the abundance of total ammonia oxidizers reached 5.7 ± 2.0 × 10⁷ cells/mL, approximately 3.5 times higher than in Period 1. In contrast to the 16S rRNA gene sequencing results, the qPCR data indicated that comammox *Nitrospira* dominated in Period 1 but was outcompeted by AOB during the early Period 2 (days 78–106) and subsequently regained dominance after day 107. This discrepancy between qPCR and 16S rRNA gene sequencing may be partly attributed to differences in 16S rRNA gene copy numbers, as *Nitrospira* genomes typically contain one to three copies, while *Nitrosomonas* generally carry only one copy, as reported in the ribosomal RNA operon copy number database (27). We then examined which *Nitrospira* KTU could be related to comammox *Nitrospira* using blastn against NCBI RefSeq genome database. The results revealed that *Nitrospira* KTUs were related to *Nitrospira defluvii* and *Nitrospira tepida* (Table S3), which are classified as strict nitrite-oxidizing *Nitrospira* (28, 29). However, because 16S rRNA-based methods cannot reliably differentiate between comammox and strict nitrite-oxidizing *Nitrospira* (30), the specific *Nitrospira* KTUs corresponding to the comammox *Nitrospira* could not be determined.

The core nitrifier-associated subcommunity, defined at a correlation cutoff of 0.6, comprised only 13 KTUs with relative abundances ranging from 1.3% to 9.4%. Two KTUs belonging to class *Bacteroidia* and family *Saprospiraceae* dominated the core nitrifier-associated subcommunity (Fig. 4D). Because detailed taxonomic assignments for the 13 core KTUs could not be resolved using the QIIME 2 Naïve Bayes classifier, we conducted additional BLASTn searches against the NCBI RefSeq genome database to identify their closest reference genomes (Table S3). These analyses revealed that, except for KTUs affiliated with the genus *Sphingobium*, genus *Gemmata*, and family *Reyranellaceae*, most core KTUs exhibited sequence similarities below the genus-level threshold of 94.5% (31) to their closest genomes, suggesting that these KTUs represent novel taxa.

### Predicted functions of the core nitrifier-associated microbes

We used PICRUSt2 to predict functional genes related to nitrogen conversion and degradation of the potential pollutants in ABS wastewater for the 13 core nitrifier-associated KTUs, aiming to evaluate their roles in the WWTP and their associations with nitrifiers. The 13 core nitrifier-associated KTUs exhibited high nearest-sequenced taxon index values of >0.15 (Fig. S5), except for KTUs belonging to the family *Reyranellaceae* and genus *Gemmata*, suggesting that functional predictions based on PICRUSt2 might be less accurate for these KTUs (32).

Figure S6 presents the distribution of genes related to nitrogen conversion, including denitrification, assimilatory nitrate reduction, dissimilatory nitrate reduction, and organic nitrogen degradation, in the 13 core KTUs. Among these KTUs, nine were predicted to encode genes associated with nitrogen cycling processes (Fig. S6), while the remaining five did not, suggesting potential interactions apart from cross-feeding of nitrogenous substrates between nitrifiers and the core KTUs. Specifically, five KTUs belonging to the genus *Sphingobium*, genus *Gemmata*, genus *Defluviicoccus*, family *Reyranellaceae*, and family *Pirellulaceae* were predicted to harbor genes involved in dissimilatory and assimilatory nitrate reduction. Moreover, denitrification-related genes were predicted in six KTUs affiliated with order *Chitinophagales*, class *Bacteroidia*, genus *Gemmata*, genus *Defluviicoccus*, family *Saprospiraceae*, and family *Reyranellaceae*.

Although detailed chemical characterization of the influent wastewater was not conducted in this study, previous investigations have reported that ABS resin manufacturing wastewater is primarily composed of nitrile compounds and aromatic derivatives. Identified constituents include diphenyl isopropanol, styrene, 2-cyanoethyl ether, 3,3´-iminobis-propanenitrile, acetophenone, 3,3´-thiodipropanenitrile, and dimethylamino-propanenitrile (33, 34). Given that the microbial catabolic pathways for many of these compounds remain poorly resolved, our analysis focused on functional genes associated with the degradation of nitriles and aromatic compounds. The primary organic nitrogen compound in the wastewater, nitrile, can be degraded by nitrilase to release ammonia (35). Alternatively, nitrile can be degraded by nitrile hydratases, releasing amides, which can be subsequently hydrolyzed by amidases to release ammonia (35). Three KTUs affiliated with genus *Sphingobium*, genus *Defluviicoccus,* and family *Reyranellaceae* were predicted to encode both nitrile hydratase and amidase, whereas one KTU classified within order *Saccharimonadales* carried *amiE* encoding amidase. These findings suggest potential roles of these taxa in nitrile decomposition (Fig. S6).

Microorganisms can aerobically degrade styrene via two primary pathways: side-chain oxygenation and direct ring cleavage (36, 37). While direct ring cleavage applies to a range of aromatic compounds, side-chain oxygenation is styrene-specific and involves *styAB* (encoding styrene monooxygenase), *styC* (styrene oxide isomerase), and *feaB* (phenylacetaldehyde dehydrogenase), which together convert styrene into phenylacetate (Fig. S7A). Among the core KTUs, only three—affiliated with genus *Gemmata*, family *Reyranellaceae*, and family *Pirellulaceae*—were predicted to harbor *feaB*, while none possessed *styAB* or *styC* (Fig. S7B). Although generally incomplete, all 13 core KTUs showed potential for phenylacetate degradation, as indicated by the KEGG module analysis (Fig. S8). Despite the limited catabolic potential of these novel taxa, side-chain oxygenation may represent a crucial route for the breakdown of styrene derivatives.

Lastly, we predicted genes for carbon cycling in the core nitrifier-associated KTUs. These KTUs generally harbored central carbon metabolic pathways, including glycolysis, gluconeogenesis, the tricarboxylic acid cycle, and the pentose phosphate pathway (Fig. S8). Most were also predicted to carry genes for glycogen biosynthesis, and several encoded genes for polyhydroxyalkanoate and trehalose biosynthesis, indicating potential for carbon and energy storage to cope with fluctuating environmental conditions such as salinity and temperature (38, 39). Additionally, genes encoding carbohydrate-active enzymes, such as glycoside hydrolases, glycosyltransferases, and carbohydrate esterases, were commonly detected in the core KTUs (Fig. S9), suggesting that these taxa might contribute to organic carbon degradation in the WWTP.

### Environmental variables and dynamics of the total community and subcommunities

**Fig 5:**
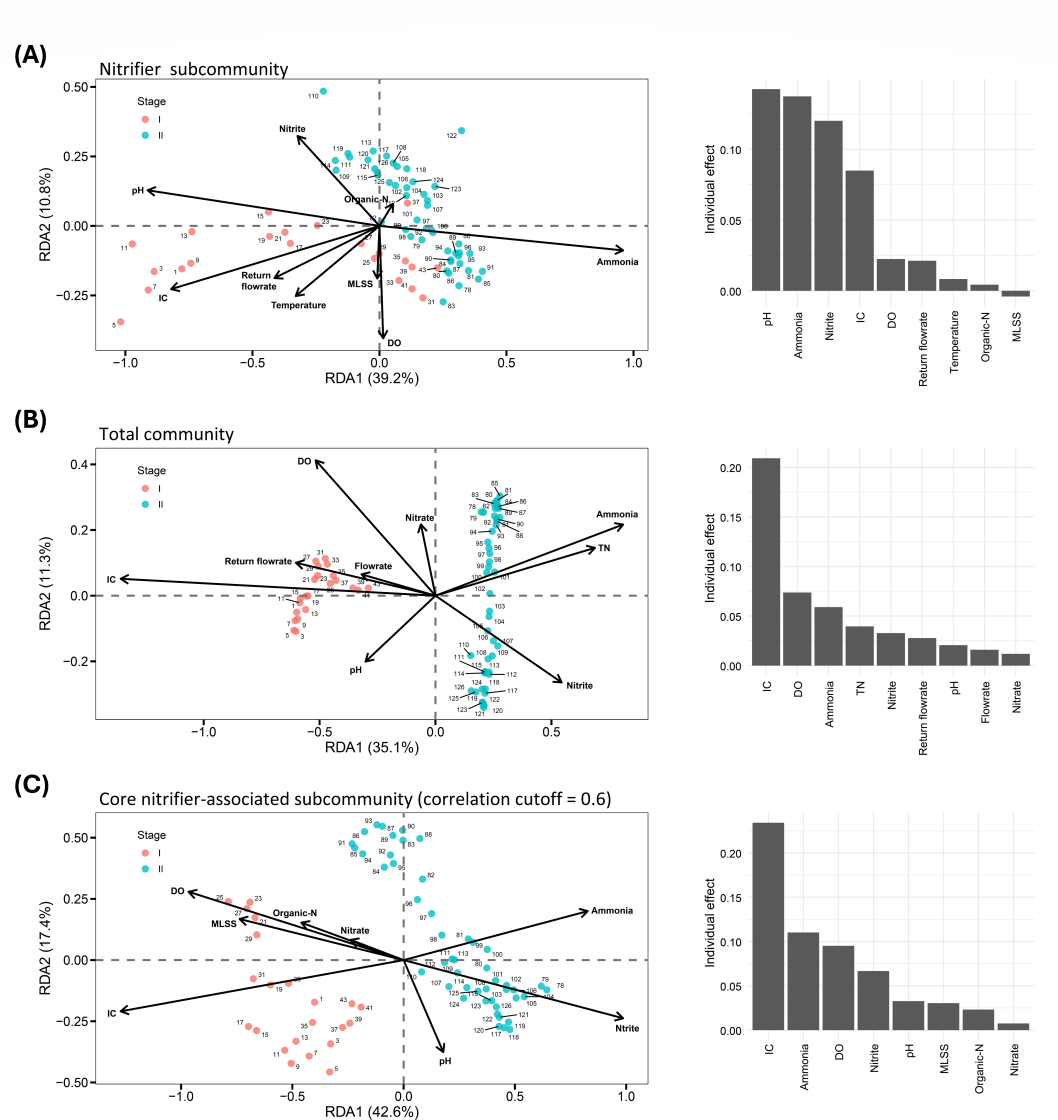
Distance-based redundancy analysis ordination plots. Left panel: distance-based redundancy analysis ordination plots (scaling 1) illustrating the relationships between a subset of environmental variables and the composition of different microbial communities. Right panel: the effects of these environmental variables on each community. The plots display the following: (**A**) the nitrifier subcommunity, (**B**) the total community, and (**C**) the core nitrifier-associated subcommunity with a correlation cutoff of 0.6. For each community, the subset of environmental variables was selected on the basis of the best Akaike information criterion scores. Organic-N, organic nitrogen; DO, dissolved oxygen; MLSS, mixed liquor suspended solid.

Given the variability in environmental conditions observed in the studied WWTP (Table 1), we investigated the influence of environmental factors on microbial community assembly using distance-based redundancy analysis (db-RDA). The best db-RDA models explained 55.4%, 61.7%, and 54.1% of the variation in the composition of the total community, nitrifier subcommunity, and core nitrifier-associated subcommunity, respectively. These findings suggest that environmental filtering was the primary driver influencing community assembly. As depicted in Fig. 5A, the dynamics of the nitrifier subcommunity was linked to high IC levels and pH in Period 1 and increased NH_4_^+^ in Period 2, with NO_2_^−^ concentrations further influencing Period 2. These four environmental factors, all exhibiting individual effects greater than 0.05, were identified as the main drivers of the variations in the nitrifier subcommunity. Because AOB and comammox *Nitrospira* alternately dominated Period 2, we further investigated which environmental factors might influence their abundance using Spearman’s rank correlation (*n* = 48) (Fig. S10). AOB abundance was positively correlated with total nitrogen, ammonium, and salinity, with correlation coefficients (ρ) of 0.66, 0.58, and 0.52, respectively. In contrast, comammox *Nitrospira* abundance showed strong negative correlations with ammonium (ρ = –0.67) and salinity (ρ = –0.41). These opposing correlations with ammonium and salinity suggest that variations in these two environmental factors were likely key drivers of the alternating dominance between AOB and comammox *Nitrospira* in Period 2.

Sample separation in the total community and core nitrifier-associated subcommunities aligned with high IC levels in Period 1 and NH_4_^+^ in Period 2 (Fig. 5B and C). Among the examined environmental factors, IC concentration played a particularly prominent role in shaping community composition, explaining 20.9% and 23.4% of the variation in the total community and the core nitrifier-associated subcommunity, respectively. Interestingly, IC concentration accounted for only 8.5% of the variation in the nitrifier subcommunity. For the total community, sample points from early Period 2 were primarily associated with dissolved oxygen, ammonia, and total nitrogen concentrations, whereas sample points from late Period 2 were positively associated with nitrite concentrations (Fig. 5B). For the core nitrifier-associated subcommunity, dissolved oxygen was the dominant environmental factor positively influencing the sample point distribution in early Period 2, while nitrite became the primary driver in the late Period 2 (Fig. 5C). Collectively, these findings indicate that the total community and the core nitrifier-associated subcommunity were more dynamic than the nitrifier subcommunity, with shifts in community composition being distinctly influenced by different environmental conditions over time.

## DISCUSSION

Consistent with previous studies (14, 15), the composition of the nitrifier subcommunity correlated more strongly with nitrification performance than that of the total community across all samples (Fig. 3F). By delineating the nitrifier-associated subcommunity, we substantially improved the predictive capacity of community diversity metrics for nitrification performance. Our results indicate that the diversity of taxa closely associated with nitrifiers explained a larger proportion of the variation in nitrification rates than did either the total community or the nitrifier subcommunity (Fig. 3D through F). Filtering out taxa with weak associations further enhanced the explanatory power of these targeted subcommunities (Fig. 3D through F), suggesting that the community structure tightly associated with functional guilds provides critical insight into ecosystem function. Importantly, changes in the composition of the nitrifier-associated subcommunity ranked among the top 3 predictors of nitrification rates, alongside influent concentrations of organic and total nitrogen (Fig. 3G). This aligns with reactor performance, which maintained >95% nitrification efficiency across sampling periods (Fig. 2A), suggesting that the influent nitrogen load was nearly equivalent to the nitrification rate. The high efficiency observed across various influent characteristics and operational parameters (Table 1; Fig. S2) underscores the resilience of the microbial community. Notably, the diversity of the nitrifier-associated subcommunity outperformed not only the nitrifier abundance and diversity but also traditional operational parameters in predicting nitrification performance (Fig. 3G). These findings refine our understanding of community diversity-function relationships in “narrow” biogeochemical processes, which have historically been attributed primarily to the diversity of functional groups (7). Our results suggest that non-nitrifying taxa, when closely associated with nitrifiers, may play pivotal roles in modulating nitrification performance, highlighting the importance of microbial network context in shaping ecosystem functions traditionally attributed to narrowly defined functional groups.

Our findings indicate that changes in the composition of the nitrifier-associated subcommunity have a greater impact on nitrification performance than does the number of taxa present in the studied WWTP. Although overall microbial diversity was relatively stable, community richness remained a significant predictor of nitrification performance in the studied AO process, demonstrating the impact of community diversity on nitrification (Table 2). The AO process maintained stable biomass concentrations at approximately 4,000 mg/L of mixed liquor suspended solids, resulting in minor changes in diversity metrics (Table S4). Despite this stability, significant positive correlations were detected between community richness—particularly within the total community and nitrifier-associated subcommunities—and the nitrification rate (Table 2). This association may not be fully explained by functional redundancy, where multiple taxa can perform overlapping metabolic functions, buffering ecosystem processes against environmental fluctuations (3, 40). In processes mediated by phylogenetically diverse taxa, greater taxonomic richness often enhances functional redundancy. However, in narrower processes, such as nitrification, taxonomic and functional diversity may be decoupled (40), and increased richness does not necessarily lead to improved functional performance. In our study, the alpha diversity of nitrifier subcommunities remained consistent across both periods (Table S4) and did not explain variations in the nitrification rate (Table 2). Instead, the observed positive relationship likely stems from redundancy among a broader group of nitrifier-associated KTUs, rather than from the nitrifiers themselves. This is consistent with the insurance hypothesis, which posits that higher biodiversity enhances ecosystem resilience by increasing the likelihood that some taxa will maintain functionality under variable conditions (41). Thus, in the studied WWTP, characterized by high organic nitrogen loads and an elevated COD:TN ratio (Table 1), overall community richness may support stable nitrification by preserving a phylogenetically diverse pool of nitrifier-associated KTUs. These taxa likely contribute to critical interactions that support nitrifier activity under fluctuating environmental conditions.

Community compositional dynamics may provide functional insurance by promoting the temporal turnover of taxa that support ecosystem processes (42, 43). In this study, compositional changes within nitrifier-associated subcommunities accounted for the largest proportion of variation in nitrification rates among all community groupings (Fig. 3). This suggests that community dynamics conferred insurance not directly to nitrifiers themselves, but to the associated taxa that potentially support their activity. The influence of community dynamics on ecosystem functioning may depend on the underlying processes of community assembly (44). Recent evidence from marine bacterioplankton systems indicates that deterministic assembly, such as environmental filtering, can amplify the functional consequences of community composition by selecting taxa with higher resource use efficiency and environmental fitness (45, 46). Consistent with this, in our system, influent characteristics and reactor operational parameters explained 50%–60% of the variation in microbial community composition, indicating a strong role of deterministic processes. These findings suggest that environmental filtering has shaped microbial assembly in the studied WWTP, likely favoring consortia that are best functionally adapted to the prevailing conditions.

The shift from AOB to comammox *Nitrospira* dominance in Period 2 was strongly correlated with influent ammonium concentration (Fig. S10). This observation aligns with previous findings that elevated influent ammonium concentrations and loadings favor the growth of AOB over comammox *Nitrospira* (47, 48). In addition, salinity was also strongly correlated with the dynamics of both AOB and comammox *Nitrospira* (Fig. S10). Salinity was notably higher in early Period 2 (1.81 ± 0.33‰) compared to Period 1 (1.70 ± 0.20‰; *P* = 0.005, Wilcoxon test) and late Period 2 (1.51 ± 0.24‰; *P* = 0.0003, Wilcoxon test). Comammox *Nitrospira* have been shown to be more sensitive to salinity than AOA and AOB (49). Another study validated the inhibitory effects of salinity on comammox *Nitrospira inopinata*, reporting an optimal growth at low salinity (0.1%–0.5‰) but complete growth inhibition at high salinity (12.8%–23.3‰) (50). Taken together, the elevated ammonium concentrations and salinity in early Period 2 likely contributed to the predominance of AOB over comammox *Nitrospira*.

Nitrifier-associated KTUs may influence nitrification performance by interacting with nitrifying bacteria. In this study, we investigated the potential associations between the 13 core nitrifier-associated KTUs and nitrifiers based on functional predictions using PICRUSt2 (Fig. S5 to S9). Although approximately 50% of the TN in the influent originated from organic nitrogen (Table 1), these 13 core KTUs could associate with nitrifiers through pathways beyond simply converting organic nitrogen to ammonia. The composition of the 13 core KTUs showed a weak correlation with the organic nitrogen loadings in the influent (adjusted *R*^2^ = 0.122, *P* = 0.009), and only four KTUs belonging to genera *Sphingobium* and *Defluviicoccus,* family *Reyranellaceae,* as well as order *Saccharimonadales* were potentially involved in nitrile degradation to ammonia. Additionally, KTUs in the genera *Sphingobium*, *Defluviicoccus*, and *Gemmata*, together with the families *Reyranellaceae* and *Pirellulaceae*, may reduce nitrate, supplying additional nitrite or ammonia that could support nitrifying bacteria. These results suggest the potential existence of a nitrite loop in the studied AO system, where heterotrophic bacteria reduce nitrate to nitrite and subsequently supply additional nitrite for nitrite-oxidizing bacteria (51). This hypothesis is supported by the high ratio of nitrite-oxidizing bacteria to ammonia-oxidizing bacteria observed in this study (Fig. S4) (52).

The core KTUs may influence nitrifiers in ways beyond nitrogen cycling. ABS resin manufacturing wastewater contains various aromatic compounds that may inhibit nitrification (53). Several core nitrifier-associated KTUs were predicted to harbor genes involved in aromatic compound degradation (Fig. S7 and S8), potentially mitigating aromatic toxicity on nitrifiers. In return, nitrifiers supply nitrate, which supports partial denitrification and dissimilatory nitrate reduction to ammonium by the core KTUs, indicating a metabolic interdependency between nitrifiers and the core nitrifier-associated KTUs (54). Furthermore, many core KTUs exhibit the genetic potential for carbohydrate degradation (Fig. S9), potentially releasing simpler organic metabolites and CO_2_. Studies have demonstrated that coculturing nitrifiers with heterotrophic bacteria can promote nitrifier growth, as heterotrophs may supply amino acids or other unidentified growth factors (55, 56). Notably, two core KTUs, affiliated with order *Saccharimonadales* and genus *Parcubacteria*, belong to candidate phyla radiation (CPR; also known as phylum *Candidatus* Patescibacteria; Table S3). CPR bacteria are characterized by ultrasmall cell sizes and streamlined genomes and are generally considered to live in parasitic or symbiotic lifestyles (57). In activated sludge systems, predicted hosts of *Saccharimonadales* are primarily members of classes *Bacteroidia*, *Alphaproteobacteria*, and *Actinobacteria* (58). Although our analysis suggests potential associations between *Saccharimonadales* and nitrifying bacteria, whether nitrifiers can serve as their hosts remains to be determined. Additionally, other types of interactions, such as predation and phage-host interaction, have also been reported in associations with nitrifying bacteria (59, 60) and may influence nitrification in WWTPs (61, 62). Although the 13 core KTUs identified in this study were observed in a full-scale WWTP treating ABS wastewater, closely affiliated taxa have also been reported across a variety of WWTPs (63–66). Moreover, closely related taxa of many core KTUs were also predicted to be associated with nitrifiers in other WWTPs (19, 62). Together, these findings suggest that the 13 core KTUs identified in our study may have broader ecological significance across diverse WWTPs. This study highlights the importance of microbial interactions in supporting nitrification and encourages future research to expand the focus from nitrifiers alone to also include their interacting partners.

Despite the novel findings in this study, two limitations should be acknowledged. First, the associations identified using SparCC, which measures linear relationships between log-transformed abundance data, do not directly reflect species interactions. This approach relies on co-occurrence patterns, which may also capture signals from environmental variations, multispecies interactions, or indirect interactions (67). Second, microbial functions were predicted using PICRUSt2 rather than via the direct reconstruction of draft genomes through metagenomics. For the nitrifier subcommunity, we selected KTUs predicted to harbor nitrification-related genes and that belong to known nitrifying genera. Although this approach ensures the reliability of selecting known nitrifying bacteria, it may overlook novel nitrifying taxa or microorganisms that do not oxidize ammonia through the conventional nitrification process, such as those involved in heterotrophic nitrification (68). Furthermore, the limited understanding of uncultured core nitrifier-associated KTUs constrains the depth of interpretation regarding their potential influence on nitrification. Nevertheless, 13 KTUs were strongly associated with nitrifiers, regardless of changes in environmental conditions. Further research using advanced methodologies is required to elucidate their precise roles in nitrification and potential interactions with nitrifiers. For culture-independent approaches, metagenomics can be employed to determine the functional potential of these KTUs. In addition, novel network inference methods, such as the multiview distance-regularized S-map, have been developed to reconstruct time-varying interaction networks and identify causal relationships between microbial taxa (69, 70). These observed interactions can then be validated using culture-based approaches, such as pure cultures or defined microbial communities. Notably, identifying microbial interactions with nitrifiers was not the primary objective of this study. Instead, the aim was to explore the relationship between microbial biodiversity and nitrification performance. This relationship could be refined by considering associations with nitrifiers, which would allow for the exclusion of taxa that are either not associated or weakly associated with nitrifiers.

This study enhances the current understanding of the relationship between biodiversity and nitrification by incorporating species co-occurrence associations. The findings emphasize the limitation of focusing solely on nitrifying microorganisms when managing nitrification in the complex and dynamic environments of wastewater treatment ecosystems, where nitrification failure has been observed despite a stable nitrifier community (71). In this study, both the diversity and dynamics of the nitrifier subcommunity remained relatively stable compared with the nitrifier-associated subcommunity (Table S4; Fig. 5). The diversity and dynamics of the nitrifier-associated subcommunity proved particularly crucial for treating industrial wastewater, which contains substantial organic nitrogen content and experiences large fluctuations in influent characteristics. In this context, the most adapted nitrifiers remained dominant over time, interacting with diverse heterotrophic populations, thereby ensuring consistently high nitrification efficiency despite changing environmental conditions and variable influent composition. Although this study is centered on a specific type of WWTP, numerous other studies have documented the potential interactions between nitrifiers and coexisting microbial communities across a range of WWTPs and environmental settings (18, 19, 66, 72, 73). These findings highlight the crucial role that the diversity of nitrifier-associated microbes plays in supporting nitrification across diverse ecosystems. For WWTPs operating under dynamic conditions, maintaining a high level of microbial diversity within the total community is essential, as this increases the likelihood of harboring a diverse set of nitrifier-associated taxa, which contributes to the overall performance and stability of nitrification. In summary, our study expands the existing framework linking biodiversity and nitrification by emphasizing the significance of broader biodiversity in WWTP ecosystems, rather than just the diversity of nitrifying microorganisms. These insights could inform improved management strategies for WWTPs, offering a predictive understanding of the relationship between microbial community composition and nitrification performance.

## MATERIALS AND METHODS

### WWTP operation and sampling

In 2018, daily time-series sampling was conducted to collect detailed physicochemical and microbial data from the biological unit of a full-scale WWTP in Tainan, Taiwan. This unit treats wastewater generated from acrylonitrile-butadiene-styrene manufacturing using an AO activated sludge process. The AO activated sludge process comprises four anoxic tanks, four oxic tanks, and a settling tank in sequence (Fig. S1), with a total working volume of 18,135 m³. The mixed liquor in the fourth oxic tank (O4) was recirculated to the first anoxic tank (A1) at a flow rate seven times that of the influent. In addition, the settled sludge was returned to the A1 tank. The hydraulic retention time of the AO activated sludge process ranged from 5.1 to 8.4 days, and the sludge retention time ranged from 45 to 55 days.

The sampling campaign was conducted in two distinct periods: Period 1 (1 May to 13 June) and Period 2 (17 July to 3 September), generating two independent data sets. Operational parameters, namely temperature, pH, dissolved oxygen, influent and sludge return flow rates, and mixed liquor suspended solid concentrations for both the AO process and the returned sludge post-settlement, were recorded by WWTP operators. Additionally, influent and effluent COD concentrations were monitored. Laboratory analyses were performed to determine influent and effluent characteristics, including total organic carbon, IC, ammonium, nitrite, nitrate, TN, total dissolved solids, conductivity, and salinity, following the protocols detailed in the Supplementary Methods (S1.2). Calculations for nitrification efficiency and nitrification rate are also detailed in the Supplementary Methods (S1.3). A summary of operational parameters and influent wastewater characteristics is presented in Table 1.

### DNA extraction, quantitative polymerase chain reaction, and amplicon sequencing

Mixed liquor samples were collected from the fourth oxic tank for microbial analysis. A 1 mL aliquot of each sample was filtered through a 0.22 µm membrane to capture microbial cells, after which total DNA was extracted using a DNeasy PowerSoil kit (Qiagen, Hilden, Germany) following the manufacturer’s instructions. A quantitative polymerase chain reaction method was then performed to determine the concentrations of bacterial 16S rRNA genes (primer set, 27F/518R [74]) and nitrification-related genes, including *amoA* genes of AOB (primer set, *amoA* 1F/2R [75]), comammox *Nitrospira* (primer set, comamoAF_mod /comamoAR_mod [48]), AOA (primer set, arch-amoA F/R [76]), and *nxrB* gene of *Nitrospira* (primer set, nxrBq F/R [77]), as previously described (47).

To characterize microbial community composition, the V3–V4 hypervariable region of the bacterial 16S rRNA gene was amplified using the primer pair 341F/806R and sequenced on the Illumina MiSeq platform. During Period 1, sequencing analysis was performed on samples collected on alternate days (e.g., Days 1, 3, 5, and 7), whereas daily samples from Period 2 were analyzed. The resulting sequences were processed to generate ASVs by using the DADA2 pipeline (78) in conjunction with the q2-dada2 plugin within QIIME 2 (v.2022.8) (79). Taxonomic classification of ASVs was performed using a Scikit-learn naïve Bayesian classifier trained on the SILVA 138 rRNA database (80) via the q2-feature-classifier plugin with default settings (81). The ASV table was rarefied 100 times at subsamples of 36,103 sequences using the q2-repeat-rarefy plugin, and the final data set represented the average values across these 100 rarefaction replicates. Next, ASVs were reclustered into KTUs using the klustering function in the KTU package with default settings (22). The KTU algorithm clusters ASVs based on their k-mer composition profiles using the partitioning around medoids algorithm, with the medoid ASV designated as the representative sequence of each cluster. The taxonomy of each KTU was assigned according to the taxonomy of its representative ASV. All downstream analyses were performed using the rarefied KTU table.

### Construction of nitrifier and nitrifier-associated subcommunities and measurement of community diversity and dynamics

Functions of KTUs were predicted using the PICRUSt2 v.2.6.2 with the PICRUSt2-SC database (82) to assign KEGG Orthology identifiers (83). Nitrifier KTUs were identified on the basis of (i) a predicted presence of *amo* or *nxr* genes and (ii) phylogenetic classification corresponding to known nitrifiers: ammonia oxidizers belonging to the genera *Nitrosomonas*, *Nitrosospira*, *Nitrosovibrio*, *Nitrosolobus*, and *Nitrosococcus* (84), as well as nitrite oxidizers belonging to the genera *Nitrobacter, Nitrotoga*, *Nitrococcus*, *Nitrospira*, *Nitrospina*, *Nitrolancea*, and *Ca*. Nitromaritima (85). KTUs meeting both criteria were selected to form the nitrifier subcommunity.

To construct microbial association networks for each sampling period, the KTU table was divided by period (Period 1: *n* = 22; Period 2: *n* = 48) and separately analyzed using the SparCC method (21) through the trans_network function in the microeco package (v.1.9.1) (86), which incorporates the SpiecEasi package (87) to perform SparCC analysis. Association networks were generated using correlation coefficient cutoffs ranging from 0.1 to 0.9 at a significance level of <0.01. Nitrifier-associated KTUs were then selected to form the nitrifier-associated subcommunity. Because separate association networks were generated for each period, a core nitrifier-associated subcommunity was further defined, comprising KTUs associated with nitrifier KTUs in both periods.

Community diversity and compositional changes in the communities were assessed using alpha and beta diversity metrics, respectively. Alpha diversity indices—including richness, Shannon, and Simpson indices—were calculated for the total community and subcommunities. Richness was defined as the number of different KTUs detected per sample, and Shannon and Simpson indices were calculated using the vegan package (88). To assess community dynamics, which quantifies variations in community structure, Bray-Curtis dissimilarity was calculated to determine pairwise differences between samples using the vegan package.

### Evaluation of the relationship between environmental variables, diversity, and nitrification

Multiple linear regression models were employed to assess the relationship between microbial community and nitrification rate. To assess the influence of community diversity, variations in richness and Shannon and Simpson indices of the total community and subcommunities were selected as predictors of nitrification rate. The impact of community composition was assessed through principal coordinates analysis to capture temporal changes. The first three axes of the principal coordinates analysis ordination, derived from Bray-Curtis dissimilarity, were incorporated into regression models to explain variations in nitrification rate.

Additionally, we assessed the importance of predictor variables in accounting for the variations in nitrification rates using random forest regression (via the randomForest package) (89). The random forest model was executed with 1,000 trees and tuned to identify the number of predictors randomly selected as candidates at each split, aiming to minimize the out-of-bag error. This model incorporates environmental variables, as outlined in Table 1, as well as microbial variables, which include variations in community composition (represented by the axes of principal coordinates analysis ordination based on Bray-Curtis dissimilarity), the abundance of bacterial 16S rRNA genes, and the abundance of *amoA* and *nxrB* genes. The importance of each predictor variable is measured by the increase in mean squared error that occurs when the respective variable is randomly permuted.

To further investigate the influence of environmental variables on microbial community dynamics, db-RDA was conducted using the environmental parameters listed in Table 1. Variables for the db-RDA models were selected using the Akaike information criterion to identify the best redundancy analysis models. Furthermore, hierarchical partitioning, implemented using the rdacca.hp package (90), was applied to quantify the individual contributions of explanatory variables in the best db-RDA models to variations in nitrification rate.

Before analysis, all variables were standardized to have a unit mean and variance. All statistical analyses, excluding those conducted in QIIME 2 and PICRUSt2, were performed using R (v.4.2.1) (91).

Between-period differences in variables were analyzed using R. Normality for each variable within each period was evaluated with the Shapiro-Wilk test. When both periods satisfied the assumption of normality, means were compared with an independent *t*-test; otherwise, the Wilcoxon rank-sum test was applied. For *t*-tests, homogeneity of variances was assessed with an F test, and either the equal-variance or unequal-variance form was used as appropriate.

## Data Availability

Sequencing data have been deposited in the NCBI Sequence Read Archive under BioProject ID PRJNA1202936.

## SUPPLEMENTAL MATERIAL

Supplemental material aem.01803-25-s0001.pdf Supplemental methods, Fig. S1 to S10, and Tables S1 to S4.

ASM does not own the copyrights to Supplemental Material that may be linked to, or accessed through, an article. The authors have granted ASM a non-exclusive, world-wide license to publish the Supplemental Material files. Please contact the corresponding author directly for reuse.
