# Supplementary material for: Microbial diversity supports nitrification: insights from a full-scale anoxic/oxic wastewater treatment process
Source: Appl Environ Microbiol. 2025 Oct 22;91(11):e01803-25. doi: 10.1128/aem.01803-25 (PMC12628772; doi:10.1128/aem.01803-25)
Supplement: Supplemental material — Supplemental methods, Fig. S1 to S10, and Tables S1 to S4. [file aem.01803-25-s0001.pdf]

# Supporting Information

# Microbial Diversity Supports Nitrification: Insights from a Full-Scale Anoxic/Oxic Wastewater Treatment Process

Yung-Hsien Shao[1], Hsiao-Pei Lu[2], Jer-Horng Wu[1]*

[1]Department of Environmental Engineering, National Cheng Kung University, Taiwan.

[2]Department of Biotechnology and Bioindustry Sciences, National Cheng Kung University, Tainan, Taiwan.

*Corresponding author
E-mail: enewujh@ncku.edu.tw
Postal address: No.1, University Road, East District, Tainan City 70101, Taiwan

**S1. Supplementary Methods** (Water quality analysis and nitrification performance calculation)

**Figure S1.** Schematic of the full-scale anoxic/oxic process in this study.

**Figure S2.** The temporal dynamics of environmental factors differed significantly between the two study periods.

**Figure S3.** Relationships between the time-lagged dynamics of core nitrifier-associated subcommunities and nitrification rates.

**Figure S4.** Variations in the abundance of bacterial 16S rRNA gene and nitrification genes.

**Figure S5.** Nearest-sequenced-taxon index of the 13 core nitrifier-associated KTUs.

**Figure S6.** Predicted nitrogen metabolism of the 13 core nitrifier-associated KTUs.

**Figure S7.** Predicted styrene degradation genes in the 13 core nitrifier-associated KTUs.

**Figure S8.** Predicted KEGG modules of the 13 core nitrifier-associated KTUs.

**Figure S9.** Predicted genes encoding Carbohydrate-active enzymes in the 13 core nitrifier-associated KTUs.

**Figure S10.** Heatmap showing Spearman's correlation between environmental factors and the abundance of AOB and comammox *Nitrospira amoA* in Period 2.

**Table S1.** Number of and sequence similarity of amplicon sequence variants re-clustered into K-mer taxonomic units.

**Table S2.** Multiple linear regression analysis of community dynamics and COD removal rate.

**Table S3.** Taxonomy and sequence similarity of nitrifier and core nitrifier-associated KTUs.

**Table S4.** Alpha diversity indices of the total community and sub-communities.

## S1. Supplementary Methods

### S1.1 Measuring influent and effluent wastewater characteristics

Concentrations of ammonium nitrogen ($NH_4^+$-N), nitrite nitrogen ($NO_2^-$-N), and nitrate-nitrogen ($NO_3^-$-N) were analyzed using Dionex ICS-1100 ion chromatographs (Thermo Fisher Scientific) with two columns: Dionex IonPac CS12A RFIC (for ammonium) and Dionex IonPac AS9-HC (for nitrite and nitrate). Total nitrogen concentration was quantified using HACH Nitrogen, Total kit (persulfate digestion method). The concentration of organic nitrogen is obtained by subtracting the concentrations of ammonium, nitrite, and nitrate nitrogen from the total nitrogen concentration.

Concentrations of total organic carbon (TOC) and inorganic carbon (IC) were analyzed using Sievers InnovOx On-Line TOC Analyzer. The conductivity, salinity, and total dissolved solids (TDS) were analyzed using a portable conductivity meter (Suntex, Taiwan).

### S1.2 Calculating nitrification performance

Nitrification performance was calculated using equations (1) to (3).

$$\text{Nitrate production} = (\text{Organic} - \text{N}_{\text{inf}} + \text{NH}_4^+{}_{\text{inf}} + \text{NO}_2^-{}_{\text{inf}}) - (\text{Organic} - \text{N}_{\text{eff}} + \text{NH}_4^+{}_{\text{eff}} + \text{NO}_2^-{}_{\text{eff}}) \quad (1)$$

$$\text{Nitrification efficiency} = \frac{\text{Nitrate production}}{(\text{Organic}-\text{N}_{\text{inf}})+(\text{NH}_4^+{}_{\text{inf}})+(\text{NO}_2^-{}_{\text{inf}})} * 100\% \quad (2)$$

$$\text{Nitrification rate} = \frac{\text{Nitrate production}}{\text{HRT}} \quad (3)$$

**Figure S1. Schematic of the anoxic/oxic (AO) process in the full-scale wastewater treatment plant.** The blue arrows indicate the flow of wastewater, while the brown arrows represent the flow of sludge.

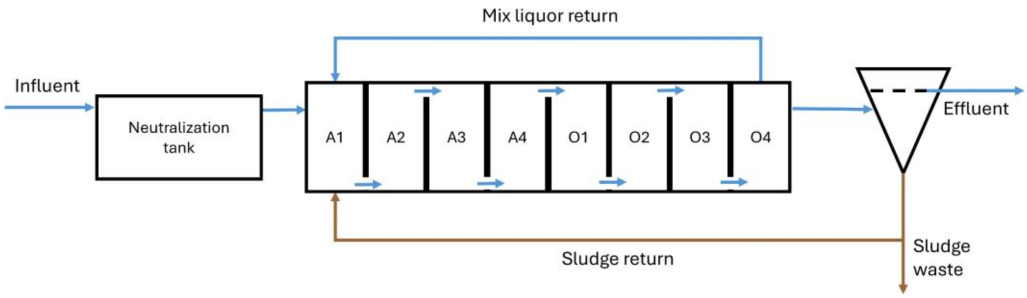

**Figure S2. The temporal dynamics of environmental factors differed significantly between the two study periods.** (A) Total nitrogen concentration, (B) ammonium concentration, (C) nitrite concentration, (D) inorganic carbon concentration, (E) temperature, (F) pH, (G) mixed liquor suspended solids (MLSS) concentration, and (H) return flowrate of settled sludge.

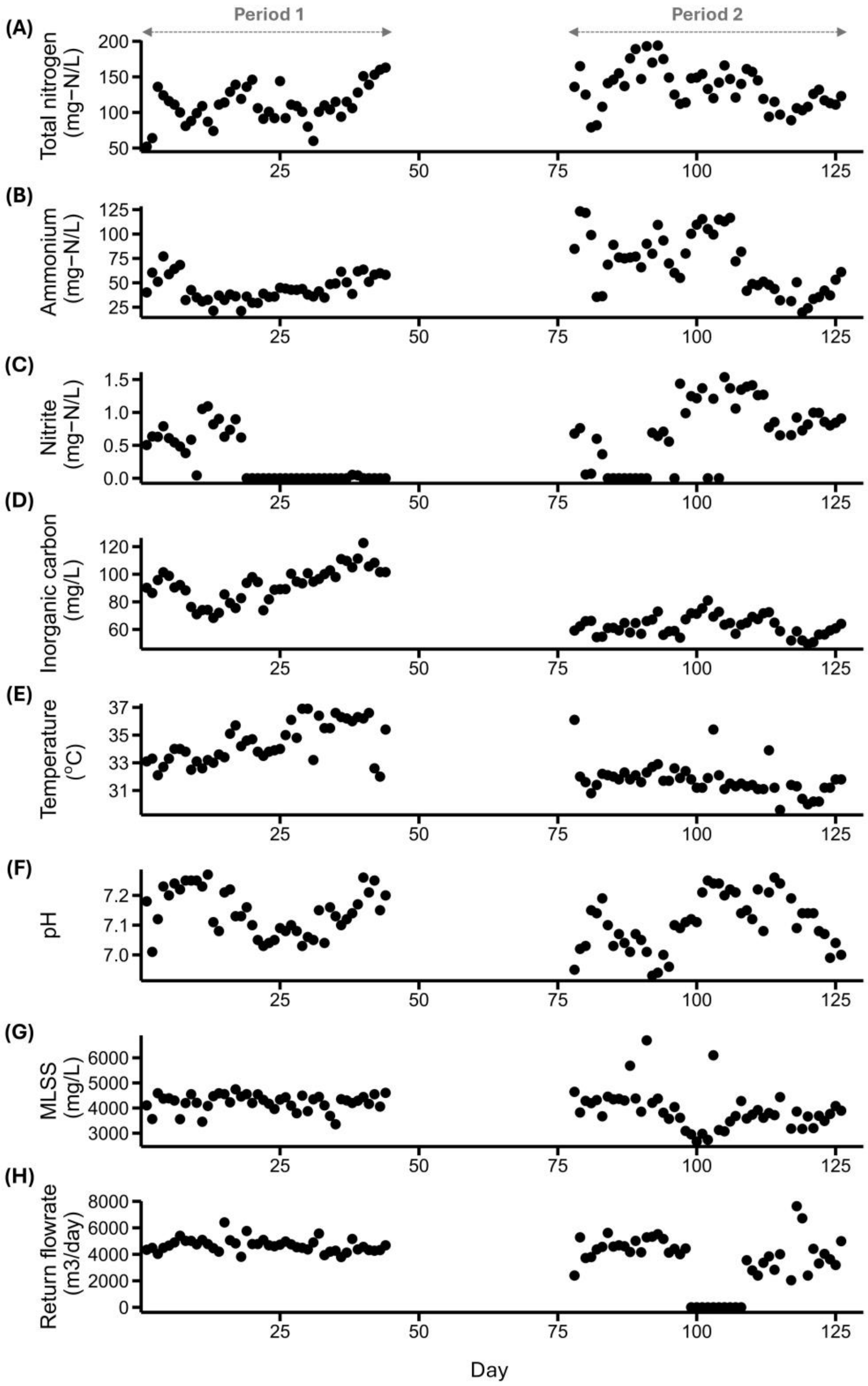

**Figure S3. Relationships between the time-lagged dynamics of core nitrifier-associated subcommunities and nitrification rates across all samples.** (A) Time lag = 2 days (n=67), (B) time lag = 4 days (n=64), (C) time lag = 6 days (n=61), and (D) time lag = 8 days (n=58). The proportion of variation in nitrification rate explained by the dynamics of core nitrifier-associated subcommunities was assessed across all samples using multiple linear regression models, with the first three axes of principal coordinate analysis derived from Bray–Curtis dissimilarity as predictors. Hollow circles indicate regression models with *p* values greater than 0.05, and solid circles indicate *p* values less than 0.05. The gray dashed line represents the variation explained by the total microbial community, and the yellow dashed line represents the variation explained by the nitrifier subcommunity.

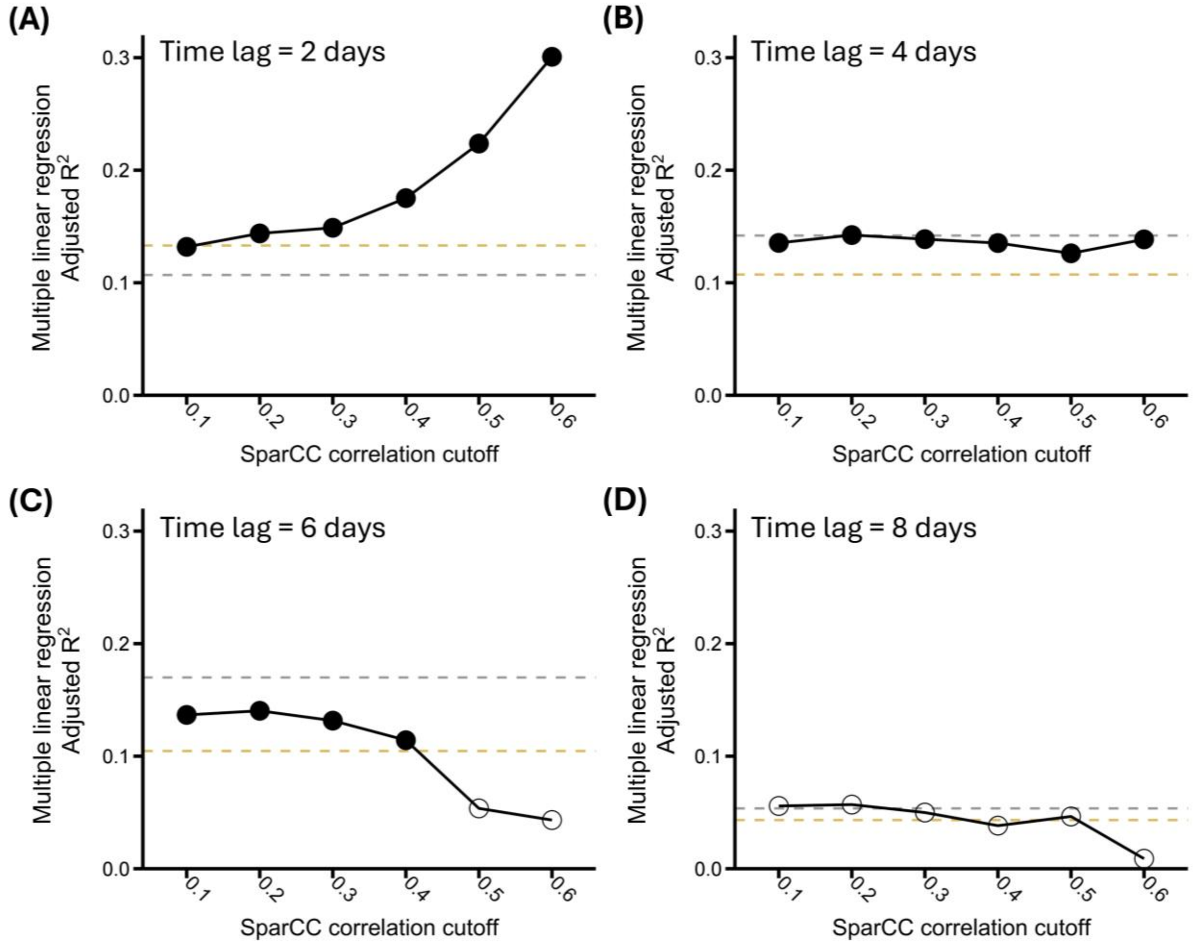

**Figure S4. Variations in the abundance of bacterial 16S rRNA gene and nitrification-related genes.** Genes encoding the ammonia monooxygenase subunit alpha (*amoA*) of ammonia-oxidizing bacteria (AOB), ammonia-oxidizing archaea (AOA), and comammox *Nitrospira* represent ammonia oxidation, while the gene encoding the nitrite oxidoreductase subunit beta (*nxrB*) of *Nitrospira* represents nitrite oxidation.

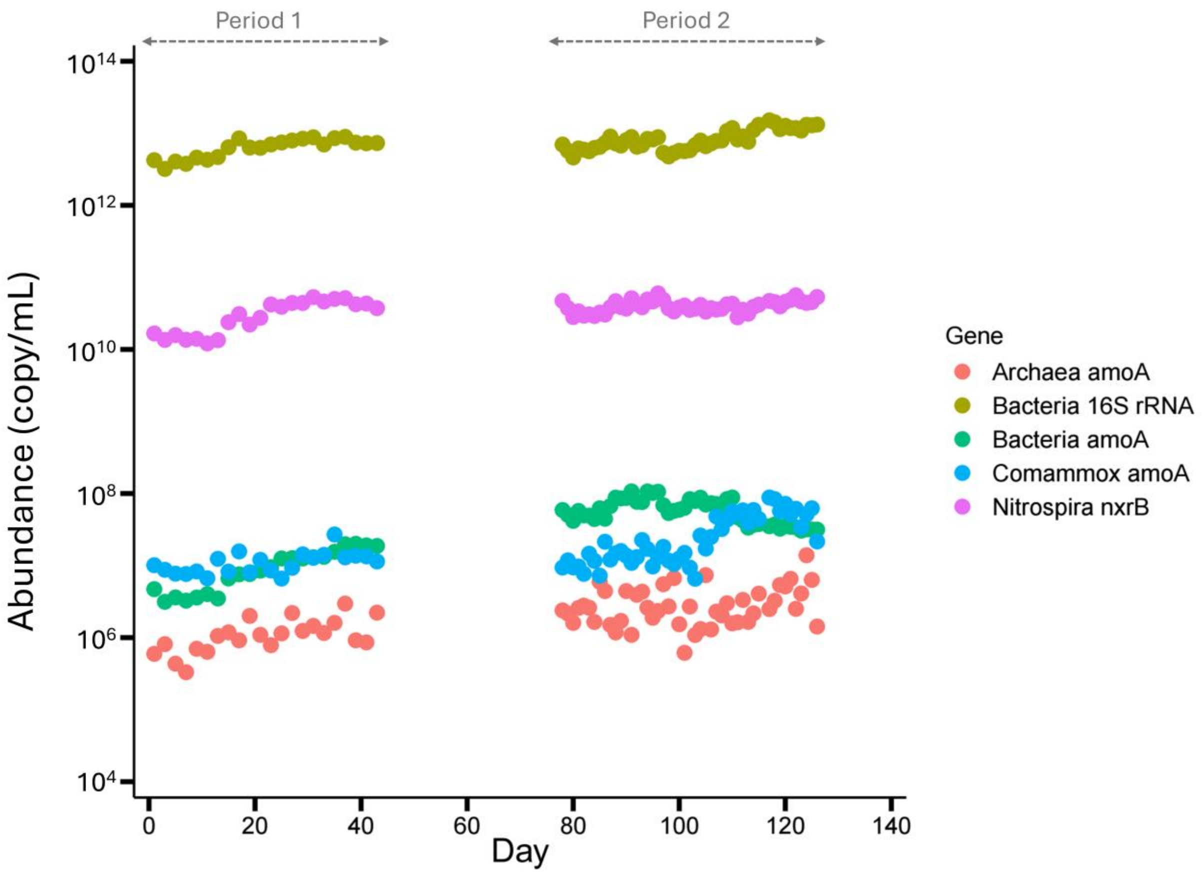

**Figure S5. Nearest-sequenced-taxon index (NSTI) of the 13 core nitrifier-associated KTUs (coefficient cutoff = 0.6).** The NSTI measures the phylogenetic distance between the query sequence and its nearest reference genome in the database. A higher NSTI value indicates that the sequence is more distantly related reference genome, suggesting less reliable functional predictions. An NSTI value below 0.15 is generally recommended for reliable functional predictions.

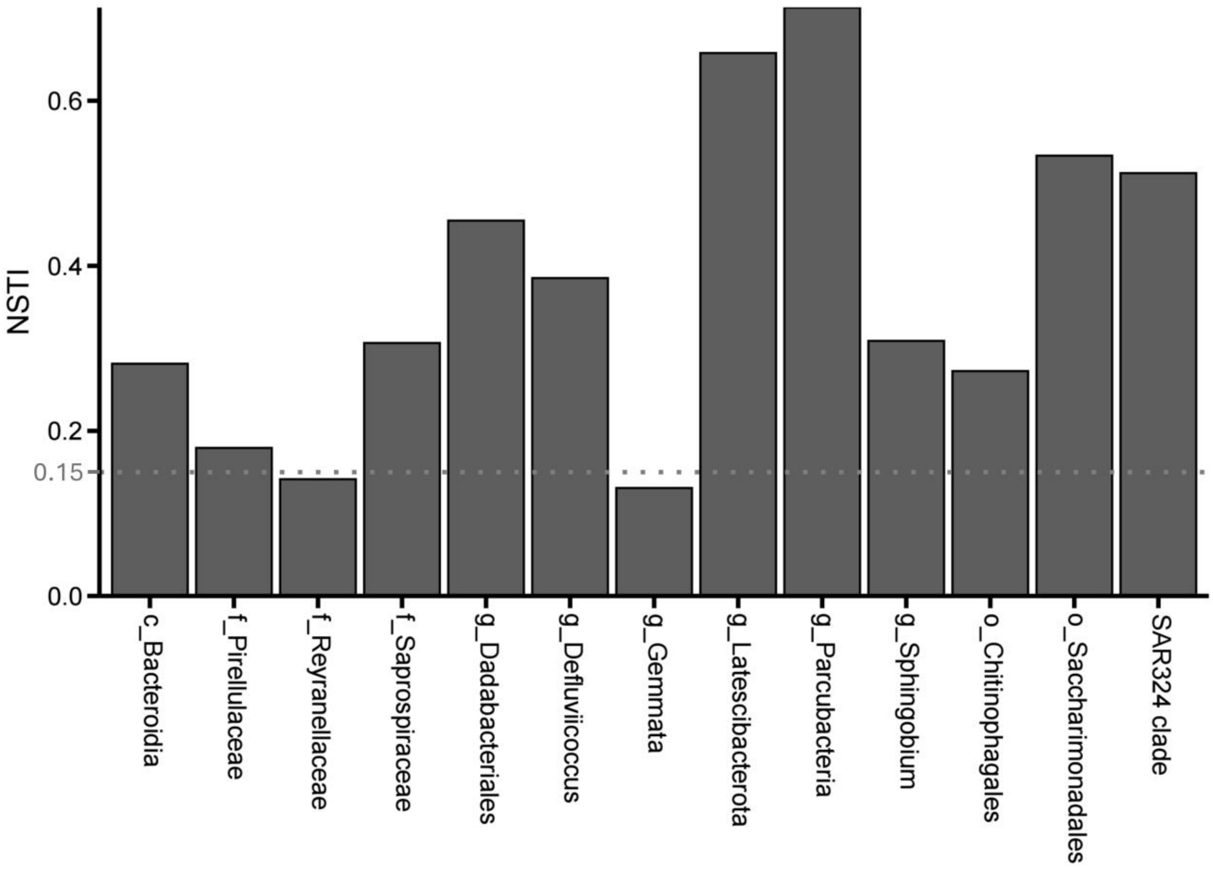

**Figure S6. Predicted nitrogen metabolism of the 13 core nitrifier-associated KTUs (coefficient cutoff 0.6).** The bubble size represents the count of predicted functional genes within the 13 KTUs. Colors indicate genes encoding enzymes involved in various nitrogen conversion pathways, including denitrification, assimilatory nitrate reduction, dissimilatory nitrate reduction, and organic nitrogen degradation.

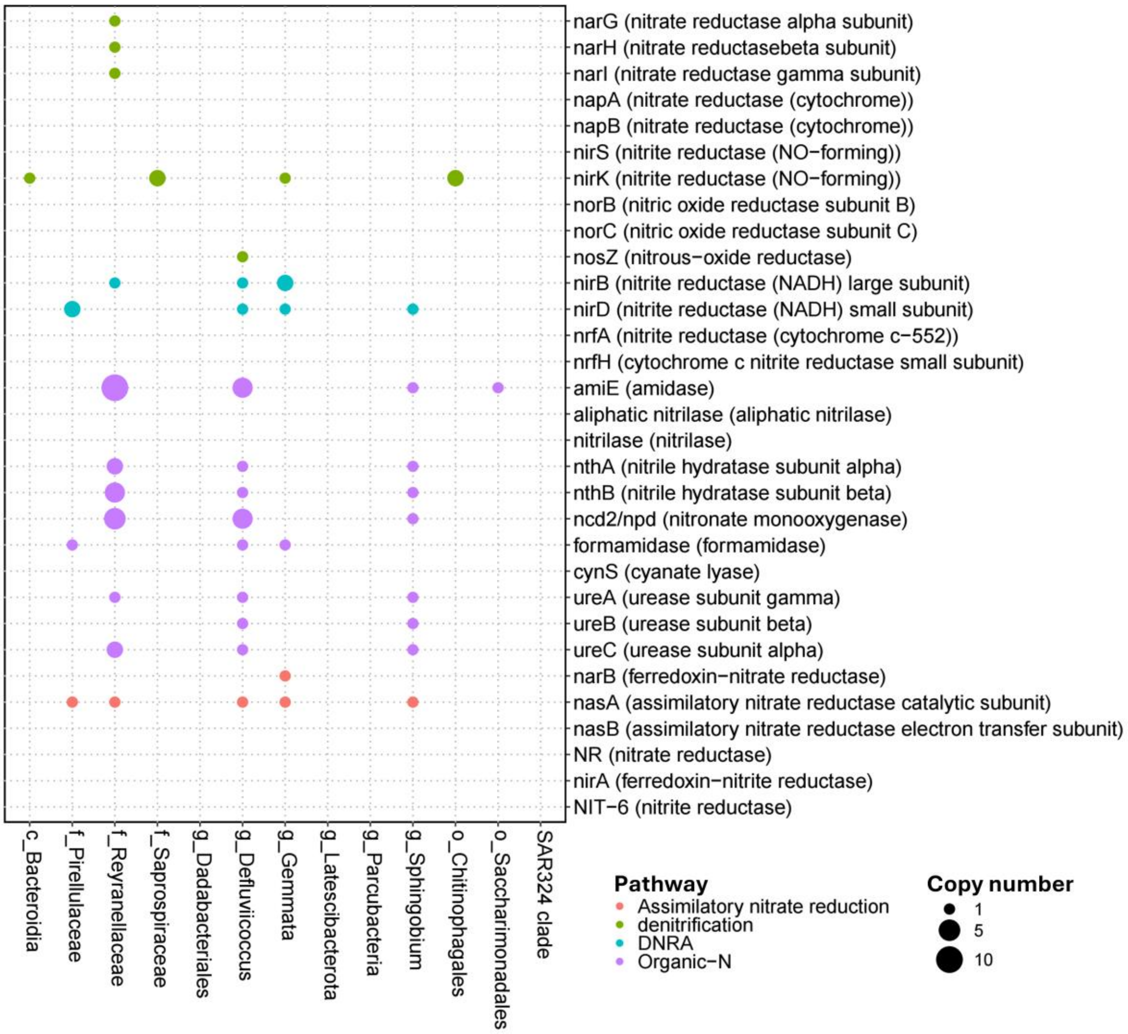

**Figure S7. Predicted styrene degradation genes in the 13 core nitrifier-associated KTUs (coefficient cutoff 0.6).** (A) Styrene degradation pathway from the KEGG database (map00643). Structural formulas were obtained from MolView (https://molview.org/). (B) Predicted genes involved in styrene degradation. Bubble size indicates the number of predicted functional genes across the 13 KTUs.

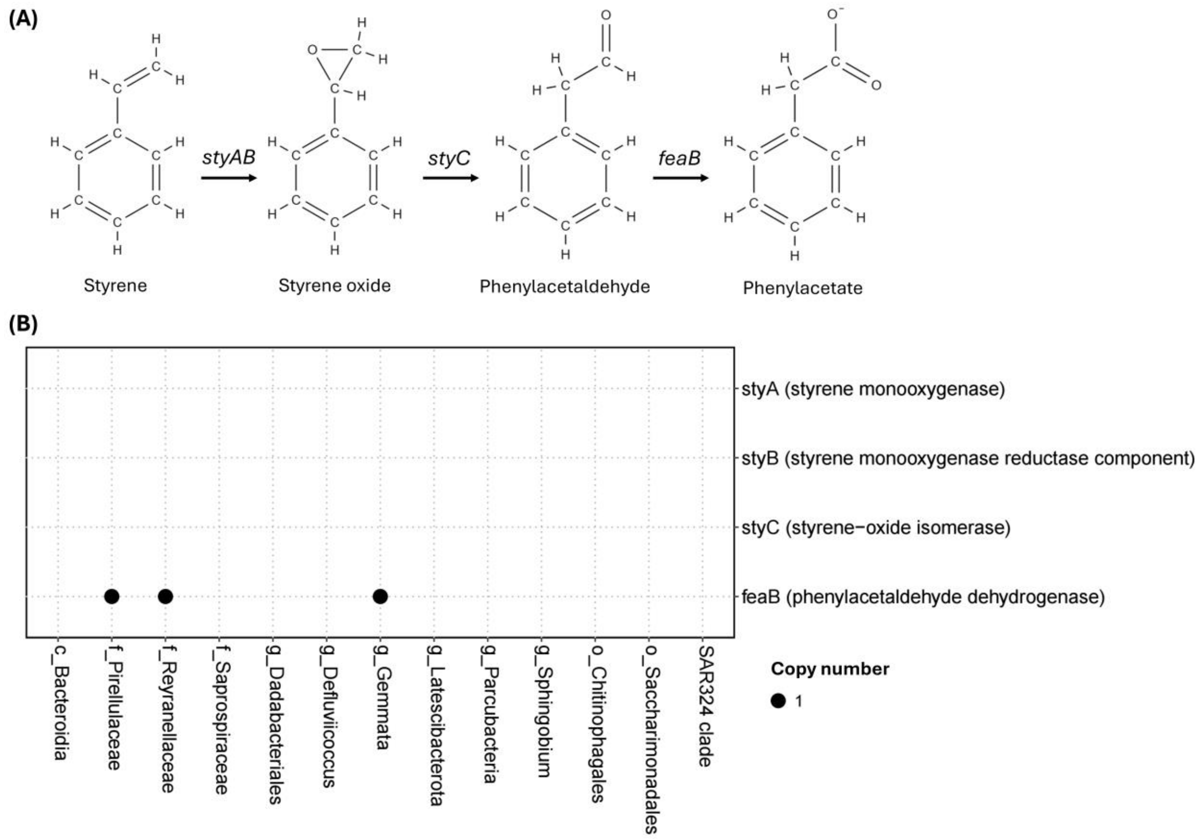

**Figure S8. Predicted KEGG modules of the 13 core nitrifier-associated KTUs (coefficient cutoff 0.6).** Colors indicate the completeness of predicted functional modules across the 13 KTUs. Polyhydroxyalkanoate (PHA) biosynthesis was predicted based on the presence or absence of *haC* encoding poly[(R)-3-hydroxyalkanoate] polymerase.

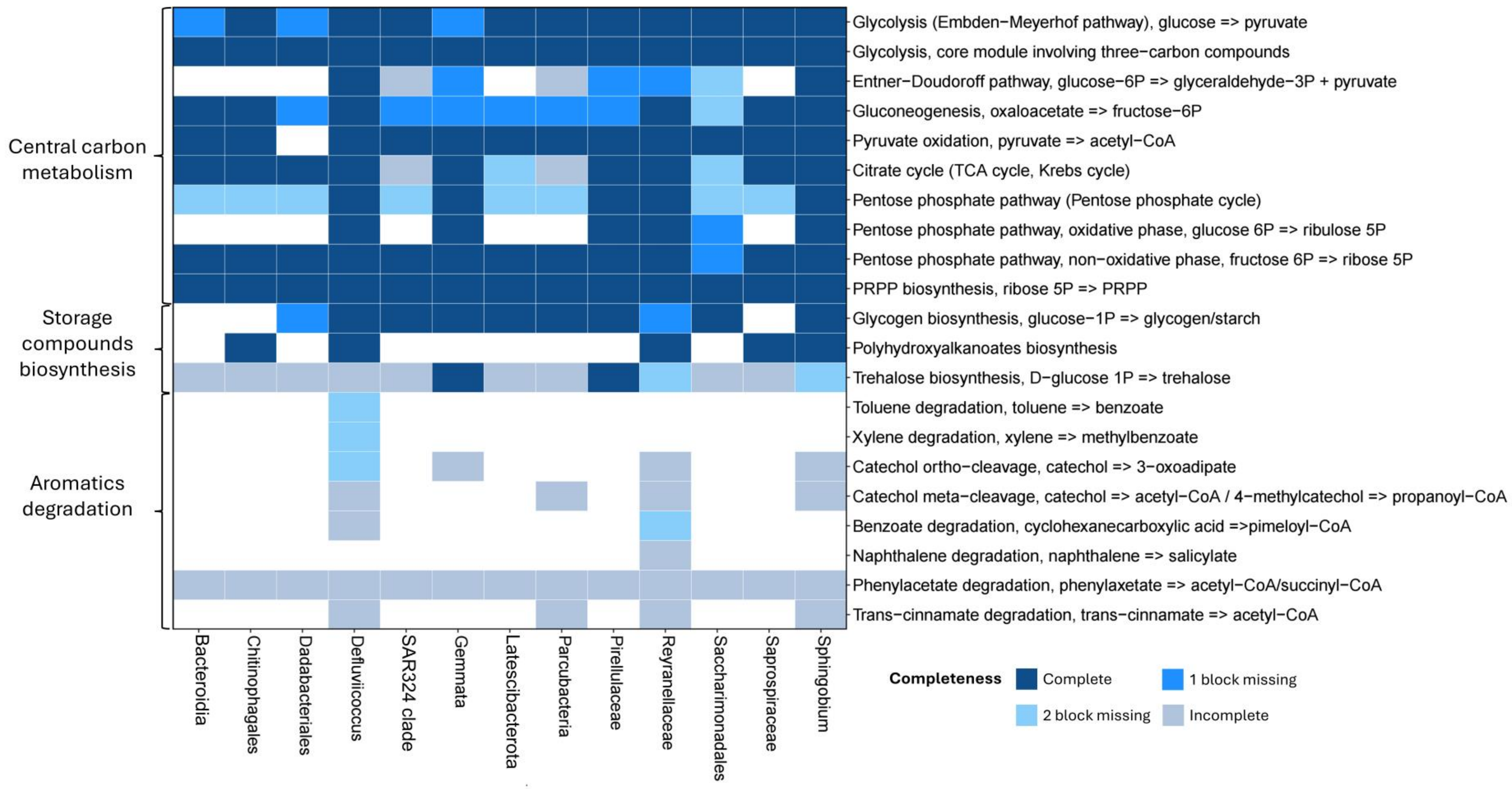

**Figure S9. Predicted genes encoding Carbohydrate-Active enZYmes (CAZys) in the 13 core nitrifier-associated KTUs (coefficient cutoff 0.6)**. Bubble size indicates the number of predicted CAZy genes across the 13 KTUs. The CAZys are divided into six categories, including Polysaccharide Lyases (PL), Glycoside Hydrolases (GH), Glycosyltransferases (GT), Carbohydrate Esterases (CE), Carbohydrate-Binding Modules (CBM), and Auxiliary Activities (AA).

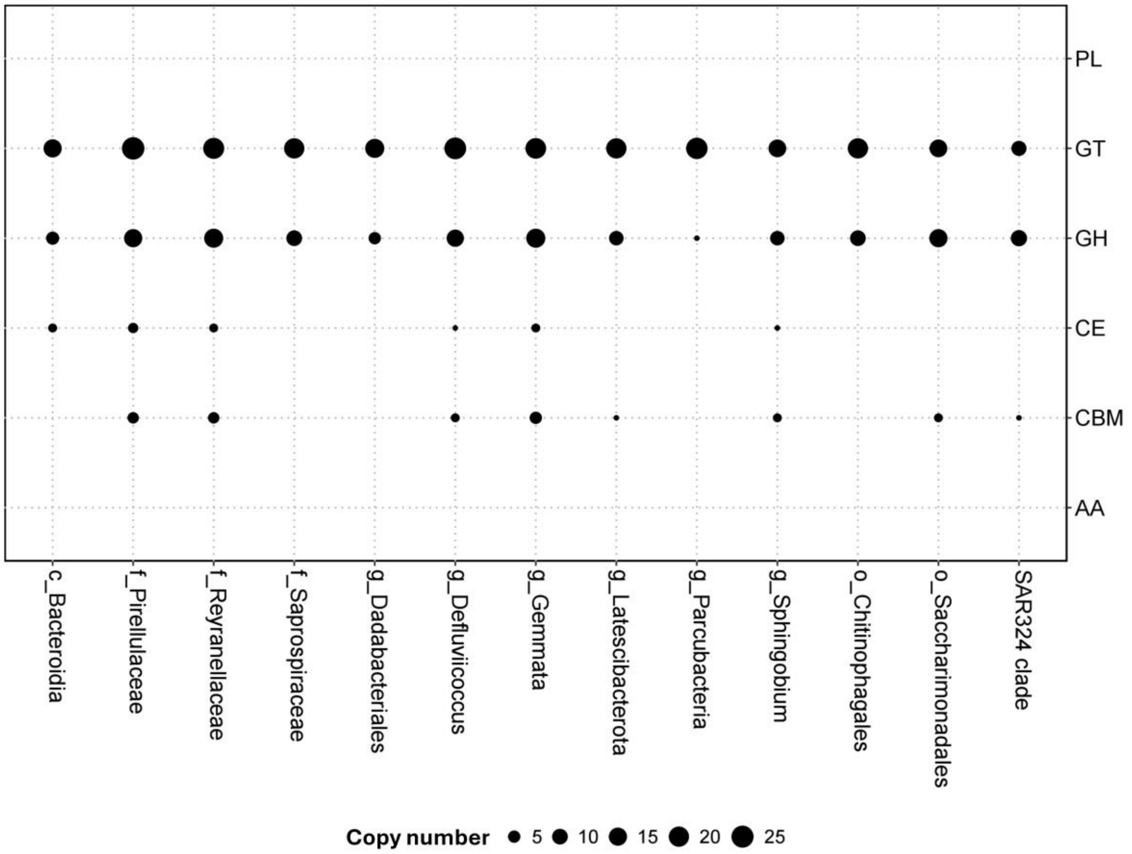

**Figure S10.** Heatmap showing Spearman's correlation between environmental factors and the abundance of AOB and comammox *Nitrospira amoA* in Period 2. Asterisks within cells indicate the level of statistical significance ($p < 0.05$, *$p < 0.01$, **$p < 0.001$).

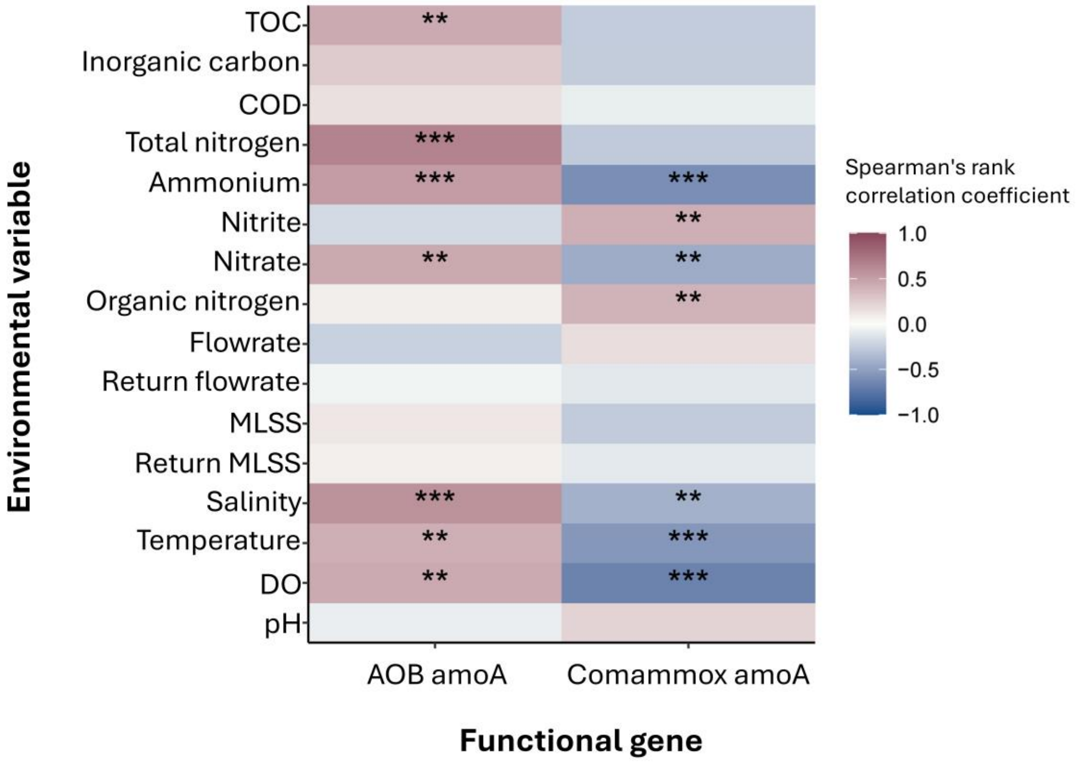

**Table S1.** Number of and sequence similarity of amplicon sequence variants (ASVs) re-clustered into K-mer taxonomic units (KTUs). For nitrifier KTUs, the average number of ASVs per KTU was 13 ± 11 with a sequence similarity of 99.63 ± 0.41%, whereas a core nitrifier-associated KTU contained an average of 14.8 ± 10.1 ASVs with a sequence similarity of 99.16 ± 1.23%.

| KTU | Subcommunity | Number of re-clustered ASVs | Sequence similarity (%) of re-clustered ASVs |
|---|---|---|---|
| *Nitrosomonas* KTU1 | Nitrifier | 26 | 99.46 |
| *Nitrosomonas* KTU2 | Nitrifier | 30 | 99.27 |
| *Nitrosomonas* KTU3 | Nitrifier | 5 | 99.72 |
| *Nitrosomonas* KTU4 | Nitrifier | 1 | 100.00 |
| *Nitrosomonas* KTU5 | Nitrifier | 1 | 100.00 |
| *Nitrosomonas* KTU6 | Nitrifier | 1 | 100.00 |
| *Nitrospira* KTU1 | Nitrifier | 16 | 99.33 |
| *Nitrospira* KTU2 | Nitrifier | 26 | 99.79 |
| *Nitrospira* KTU3 | Nitrifier | 15 | 99.84 |
| *Nitrospira* KTU4 | Nitrifier | 17 | 99.81 |
| *Nitrospira* KTU5 | Nitrifier | 5 | 98.68 |
| f_Saprospiraceae | Core nitrifier-associated | 21 | 99.85 |
| c_Bacteroidia | Core nitrifier-associated | 21 | 98.93 |
| g_Dadabacteriales | Core nitrifier-associated | 3 | 97.73 |
| f_Pirellulaceae | Core nitrifier-associated | 3 | 99.97 |
| g_Defluviicoccus | Core nitrifier-associated | 22 | 99.78 |
| g_Latescibacteria | Core nitrifier-associated | 12 | 99.88 |
| o_Chitinophagales | Core nitrifier-associated | 4 | 97.44 |
| g_Gemmata | Core nitrifier-associated | 4 | 96.22 |
| g_Parcubacteria | Core nitrifier-associated | 26 | 99.92 |
| f_Reyranellaceae | Core nitrifier-associated | 28 | 99.82 |
| o_Saccharimonadales | Core nitrifier-associated | 3 | 99.96 |
| g_Sphingobium | Core nitrifier-associated | 25 | 99.80 |
| SAR324 clade | Core nitrifier-associated | 21 | 99.82 |

**Table S2.** The dynamics of total and sub-community composition based on Bray-Curtis in explaining the COD removal rate through multiple linear regression analysis.

| Community | Predictor | COD removal rate (all samples, n=70) | |
|---|---|---|---|
| | | Standardized coefficient | Adjusted $R^2$ |
| Total community | PCoA axis 1 | -0.05 | -0.03 |
| | PCoA axis 2 | -0.08 | |
| | PCoA axis 3 | 0.08 | |
| Nitrifier sub-community | PCoA axis 1 | 0.17 | 0.02 |
| | PCoA axis 2 | -0.13 | |
| | PCoA axis 3 | 0.13 | |
| Core nitrifier-associated sub-community | PCoA axis 1 | -0.04 | -0.01 |
| | PCoA axis 2 | 0.10 | |
| | PCoA axis 3 | 0.15 | |

**Table S3.** Taxonomy and sequence similarity of nitrifier and core nitrifier-associated KTUs. Taxonomy was assigned using QIIME2 with the SILVA 138 rRNA database, and closest reference genomes were identified by BLASTn against the NCBI RefSeq genome database.

| Query | Taxonomy (SILVA database) | Closest genome (NCBI RefSeq genome database) | Query coverage (%)* | Identity (%)* |
|---|---|---|---|---|
| *Nitrospira* KTU1 | d__Bacteria; p__Nitrospirae; c__Nitrospira; o__Nitrospirales; f__Nitrospiraceae; g__Nitrospira | *Nitrospira tepida* DNF | 100 | 97.2 |
| *Nitrospira* KTU2 | d__Bacteria; p__Nitrospirae; c__Nitrospira; o__Nitrospirales; f__Nitrospiraceae; g__Nitrospira | *Nitrospira tepida* DNF | 100 | 100.0 |
| *Nitrospira* KTU3 | d__Bacteria; p__Nitrospirae; c__Nitrospira; o__Nitrospirales; f__Nitrospiraceae; g__Nitrospira; s__Nitrospira sp. OLB3 | *Nitrospira defluvii* | 100 | 97.2 |
| *Nitrospira* KTU4 | d__Bacteria; p__Nitrospirae; c__Nitrospira; o__Nitrospirales; f__Nitrospiraceae; g__Nitrospira; s__Nitrospira sp. | *Nitrospira defluvii* | 100 | 100.0 |
| *Nitrospira* KTU5 | d__Bacteria; p__Nitrospirae; c__Nitrospira; o__Nitrospirales; f__Nitrospiraceae; g__Nitrospira | *Nitrospira tepida* DNF | 100 | 97.0 |
| *Nitrosomonas* KTU1 | d__Bacteria; p__Proteobacteria; c__Gammaproteobacteria; o__Betaproteobacteriales; f__Nitrosomonadaceae; g__Nitrosomonas; s__Nitrosomonas sp. Nm132 | *Nitrosomonas* sp. Nm58 | 100 | 99.5 |
| *Nitrosomonas* KTU2 | d__Bacteria; p__Proteobacteria; c__Gammaproteobacteria; o__Betaproteobacteriales; f__Nitrosomonadaceae; g__Nitrosomonas | *Nitrosomonas* sp. RBC | 100 | 98.4 |
| *Nitrosomonas* KTU3 | d__Bacteria; p__Proteobacteria; c__Gammaproteobacteria; o__Betaproteobacteriales; f__Nitrosomonadaceae; g__Nitrosomonas | *Nitrosomonas aestuarii* Nm36 | 100 | 95.8 |
| *Nitrosomonas* KTU4 | d__Bacteria; p__Proteobacteria; c__Gammaproteobacteria; o__Betaproteobacteriales; f__Nitrosomonadaceae; g__Nitrosomonas; s__Nitrosomonas sp. Nm132 | *Nitrosomonas nitrosa* Nm90 | 100 | 89.6 |
| *Nitrosomonas* KTU5 | d__Bacteria; p__Proteobacteria; c__Gammaproteobacteria; o__Betaproteobacteriales; f__Nitrosomonadaceae; g__Nitrosomonas; s__Nitrosomonas sp. Nm132 | *Nitrosomonas nitrosa* Nm90 | 100 | 90.1 |
| *Nitrosomonas* KTU6 | d__Bacteria; p__Proteobacteria; c__Gammaproteobacteria; o__Betaproteobacteriales; f__Nitrosomonadaceae; g__Nitrosomonas; s__ | *Nitrosomonas marina* Nm22 | 100 | 87.8 |

| | | | | |
|---|---|---|---|---|
| Core nitrifier-associated KTU1 | d_Bacteria; p_Dadabacteria; c_Dadabacteriia; o_Dadabacteriales; f_Dadabacteriales; g_Dadabacteriales | *Rhodoligotrophos appendicifer* strain 120-1 | 99 | 86.7 |
| Core nitrifier-associated KTU2 | d_Bacteria; p_Patescibacteria; c_Saccharimonadia; o_Saccharimonadales | *Ca.* Nanogingivalis gingivitcus | 100 | 85.7 |
| Core nitrifier-associated KTU3 | d_Bacteria; p_Latescibacterota; c_Latescibacterota; o_Latescibacterota; f_Latescibacterota; g_Latescibacterota | *Salmonella enterica* subsp. enterica serovar Anatum4 | 100 | 93.3 |
| Core nitrifier-associated KTU4 | d_Bacteria; p_Patescibacteria; c_Parcubacteria; o_Parcubacteria; f_Parcubacteria; g_Parcubacteria | *Weissella confuse* VTT E-153457 | 100 | 90.8 |
| Core nitrifier-associated KTU5 | d_Bacteria; p_Proteobacteria; c_Alphaproteobacteria; o_Sphingomonadales; f_Sphingomonadaceae; g_Sphingobium | *Sphingobium wenxiniae* | 100 | 98.0 |
| Core nitrifier-associated KTU6 | d_Bacteria; p_Bacteroidota; c_Bacteroidia; o_Chitinophagales; f_Saprospiraceae | *Neolewinella maritima* CECT 8419 | 100 | 87.6 |
| Core nitrifier-associated KTU7 | d_Bacteria; p_Planctomycetota; c_Planctomycetes; o_Pirellulales; f_Pirellulaceae | *Bythopirellula polymerisocia* Pla144 | 100 | 87.8 |
| Core nitrifier-associated KTU8 | d_Bacteria; p_SAR324_clade; c_SAR324_clade; o_SAR324_clade; f_SAR324 | *Angustibacter luteus* JCM 17683 | 100 | 85.3 |
| Core nitrifier-associated KTU9 | d_Bacteria; p_Bacteroidota; c_Bacteroidia; o_Chitinophagales | *Deminuibacter* sp. K23C18032701 | 100 | 86.5 |
| Core nitrifier-associated KTU10 | d_Bacteria; p_Planctomycetota; c_Planctomycetes; o_Gemmatales; f_Gemmataceae; g_Gemmata | *Gemmata palustris* G18 | 100 | 95.7 |
| Core nitrifier-associated KTU11 | d_Bacteria; p_Proteobacteria; c_Alphaproteobacteria; o_Defluviicoccales; f_Defluviicoccaceae; g_Defluviicoccus | *Defluviicoccus vanus* Ben 114 | 100 | 92.9 |
| Core nitrifier-associated KTU12 | d_Bacteria; p_Bacteroidota; c_Bacteroidia | *Sphingobacterium faecale* C459-1 | 100 | 86.9 |
| Core nitrifier-associated KTU13 | d_Bacteria; p_Proteobacteria; c_Alphaproteobacteria; o_Reyranellales; f_Reyranellaceae | *Reyranella* sp. CPCC | 100 | 96.4 |

*Query coverage and identity to the closest genome identified via BLASTn against the NCBI RefSeq genome database.

**Table S4**. Alpha diversity indices of the total community and sub-communities. Normality of each variable within each period was evaluated using the Shapiro–Wilk test. Depending on the results, between-period differences were tested with either an independent *t* test (when both periods were normally distributed) or a Wilcoxon rank-sum test (when normality was not satisfied). Indices with significant differences between Stage I and Stage II ($p < 0.05$) are shown in bold.

| Community | Alpha diversity index | Value in period I (n=22) (Mean±SD) | Value in period II (n=48) (Mean±SD) | Test | *p*-value |
|---|---|---|---|---|---|
| Total community | Richness | 567±59 | 524±91 | Wilcoxon | 0.108 |
| | Shannon | 5.3806±0.1617 | 5.2813±0.1839 | Wilcoxon | 0.056 |
| | **Simpson** | **0.9900±0.0023** | **0.9876±0.0039** | **Wilcoxon** | **0.001** |
| Nitrifier subcommunity | Richness | 5±1 | 5±1 | Wilcoxon | 0.506 |
| | Shannon | 0.8602±0.1617 | 0.9129±0.1526 | Wilcoxon | 0.125 |
| | Simpson | 0.4444±0.0902 | 0.4542±0.0724 | Wilcoxon | 0.787 |
| Core nitrifier-associated subcommunity | **Richness** | **6±1** | **6±1** | **Wilcoxon** | **0.020** |
| | **Shannon** | **0.8654±0.1104** | **1.1729±0.1458** | **t-test** | **0.000** |
| | **Simpson** | **0.4498±0.0702** | **0.6166±0.0570** | **Wilcoxon** | **0.000** |
